# PROS1 released by lung basal cells limits inflammation in epithelial and monocytes during SARS-CoV-2 infection

**DOI:** 10.1093/discim/kyaf012

**Published:** 2025-08-27

**Authors:** Theodoros Simakou, Agnieszka M Szemiel, Lucy MacDonald, Karen Kerr, Domenico Somma, Katy Diallo, Jack Frew, Olympia M Hardy, Marcus Doohan, Aziza Elmesmari, Charles McSharry, Stefano Alivernini, Thomas D Otto, Arvind H Patel, Mariola Kurowska-Stolarska

**Affiliations:** School of infection and Immunity, University of Glasgow, Glasgow, UK; CVR-CRUSH, Medical Research Council, University of Glasgow Centre for Virus Research, Glasgow, UK; School of infection and Immunity, University of Glasgow, Glasgow, UK; CVR-CRUSH, Medical Research Council, University of Glasgow Centre for Virus Research, Glasgow, UK; School of infection and Immunity, University of Glasgow, Glasgow, UK; School of infection and Immunity, University of Glasgow, Glasgow, UK; School of infection and Immunity, University of Glasgow, Glasgow, UK; School of infection and Immunity, University of Glasgow, Glasgow, UK; School of infection and Immunity, University of Glasgow, Glasgow, UK; School of infection and Immunity, University of Glasgow, Glasgow, UK; School of infection and Immunity, University of Glasgow, Glasgow, UK; Immunology Research Core Facility, Gemelx1li Science and Technology Park, Fondazione Policlinico Universitario A. Gemelli IRCCS, Rome, Italy; Division of Rheumatology, Fondazione Policlinico Universitario A. Gemelli IRCCS, Rome, Italy; School of infection and Immunity, University of Glasgow, Glasgow, UK; CVR-CRUSH, Medical Research Council, University of Glasgow Centre for Virus Research, Glasgow, UK; School of infection and Immunity, University of Glasgow, Glasgow, UK

**Keywords:** PROS1, inflammation, viral, epithelial cell, macrophage

## Abstract

**Introduction:**

Factors regulating the severity of pneumonitis during viral infections remain unresolved. We previously found higher expression of protein S (PROS1) in lung epithelium of mild compared to severe coronavirus disease 2019 (COVID-19) patients. We hypothesized that PROS1 may protect the upper airways by regulating epithelial and myeloid cell responses during severe acute respiratory syndrome coronavirus 2 (SARS-CoV-2) infection.

**Methods:**

To test this, *in vitro* air–liquid interface (ALI) cultures of primary healthy human lung epithelial cells were infected with SARS-CoV-2. This model, validated through immunofluorescent staining, confocal microscopy, and single-cell RNA-sequencing, replicated pathogenic changes seen in the lungs of COVID-19. Regulation and secretion of PROS1, along with multiple soluble mediators, were quantified in control and infected cultures using ELISAs.

**Results:**

We found that PROS1 is present in the basal cells of healthy pseudostratified epithelium and is released during SARS-CoV-2 infection through an IFN-mediated process. Transcriptome analysis revealed that PROS1 downregulated the SARS-CoV-2-induced proinflammatory phenotypes of basal cells, transforming pathogenic CXCL10/11^high^ into a regenerative S100A2^pos^KRT^high^ basal cell phenotype. In parallel, SARS-CoV-2 increased the secretion of M-CSF from epithelial cells, which induced the expression of PROS1 receptor MERTK on monocytes interacting with the lung epithelium. PROS1, in turn, shifted SARS-CoV-2-induced pathogenic monocyte phenotypes toward a phenotype with increased MHC class II.

**Conclusion:**

These findings highlight the crucial role of PROS1 in protecting against severe lung pathology caused by SARS-CoV-2, by reducing epithelial- and monocyte-derived inflammation, promoting pro-repair epithelial phenotypes, and enhancing antigen presentation in myeloid cells.

## Teaser

1. SARS-CoV-2 infection of the epithelium results in the release of IFN. 2. IFN secretion has an autocrine effect on epithelial cells. 3. Infection and IFN cause the release of PROS1 from the basal cells, as well as M-CSF from the epithelium. 4-5. PROS1 acts on basal cells that express MERTK, a PROS1 receptor, and downregulates SARS-CoV-2-induced proinflammatory phenotypes while upregulating KRThigh basal cells with repair phenotypes. 6. Epithelium-derived M-CSF drives MERTK expression on infiltrating monocytes. 7. PROS1 downregulates SARS-CoV-2-induced monocyte clusters that express pro-coagulation and complement genes, while upregulating clusters with higher MHC class II expression. 8. In summary, PROS1 regulates epithelial and monocyte responses by reducing proinflammatory activation of the epithelium and promoting pro-repair phenotypes, while supporting a myeloid cell phenotype with efficient antigen presentation. Together, these effects contribute to a milder form of COVID-19. Abbreviations: BCs Basal Cells, IFN Interferon, M-CSF Macrophage Colony Stimulating Factor, MHC Major Histocompatibility Complex, KRT Cytokeratin, SARS-CoV-2 Severe Acute Respiratory Syndrome Corona Virus 2, MERTK Mer Tyrosine Kinase.

## Graphical Abstract

**Graphical Abstract:**
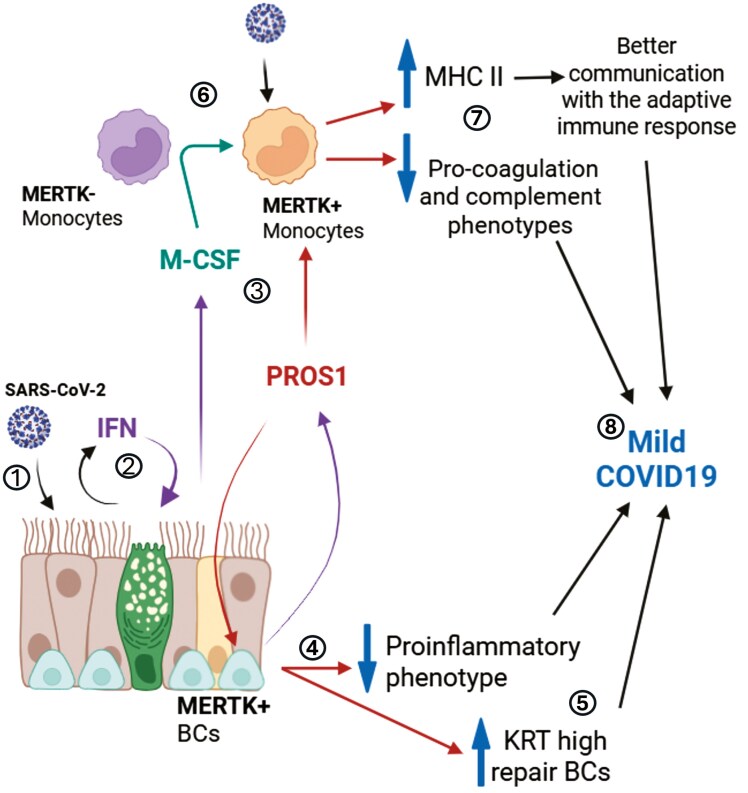

## Introduction

Before the introduction of vaccination against severe acute respiratory syndrome coronavirus 2 (SARS-CoV-2), infection and subsequent coronavirus disease 2019 (COVID-19) illness typically caused 14% of patients to develop severe disease requiring intensive care and oxygen support, while 5% of patients manifested critical disease with life-threatening pneumonia, acute respiratory distress syndrome (ARDS) or septic shock that culminated in multiorgan dysfunction and death [1, 2]. The severity and mortality of SARS-CoV-2 infection are associated with hyperinflammation and coagulopathy [3], and aberrant activation of myeloid cells in the blood [4, 5] and lung [6–8].

While vaccination has transformed the management of COVID-19, SARS-CoV-2 infection offers a valuable model for better understanding virus-induced epithelial and immune responses in the lungs. Studying this model can help dissect the cellular and molecular mechanisms that limit severe viral disease, identifying pathways that can be targeted to improve clinical outcomes.

The airway epithelium forms a first line of defence against respiratory viruses by acting as a physical barrier with tight junctions and mucociliary apparatus and by releasing multiple antiviral and proinflammatory mediators [9, 10]. The airway epithelial cells are primary portals of entry for SARS-CoV-2 due to expression of ACE2, TMPRSS2, and NRP1 [11–14]. They also express multiple pattern recognition receptors, such as the endosomal Toll-like receptors TLR3, TLR7, TLR8, and TLR9, the cytoplasmic receptors retinoic acid-inducible gene I (RIG-I) and melanoma differentiation-associated gene 5 (MDA5), and surface receptors such as TLR2, all of which allow them to detect SARS-CoV-2 and initiate the antiviral interferon (IFN) response [15, 16]. Type I and type III IFNs are crucial for the successful defence against SARS-CoV-2 and the prognosis toward mild COVID-19 [9, 17–19].

Airway epithelium infected with SARS-CoV-2 interacts with infiltrating monocytes at an early stage of disease, and this interaction determines disease severity by inducing or modulating overactivation of the innate immune system [20]. Mononuclear phagocytes account for 80% of total bronchial alveolar lavage fluid (BALF) cells in patients with severe COVID-19 compared to only 60% in patients with mild disease and 40% in healthy controls [7].

Myeloid cells can also directly interact with SARS-CoV-2. They bind spike protein using C-type lectins, leading to the induction of their proinflammatory responses [21]. It has also been shown that they serve as a reservoir of SARS-CoV-2 [22]. About 10% of monocytes and 8% of lung macrophages in patients with COVID-19 get infected with SARS-CoV-2, mostly mediated by CD16 and/or CD64 uptake of opsonized virus [22]. Overactivation of infiltrating monocytes and monocyte-derived macrophages, either by SARS-CoV-2-infected epithelium or directly by the virus, mediates cytokine shock and coagulopathy that leads to severe lung pathology [3, 6].

We have previously identified a potential mechanism that could control the severity of the immune response against SARS-CoV-2. Protein S (PROS1), an anticoagulant and anti-inflammatory mediator [23], was elevated at the mRNA level in the ciliated epithelial cells specifically in mild COVID-19 cases, but not in severe cases [24]. Interestingly, levels of PROS1 in the plasma of patients with mild or severe COVID-19 showed no difference, suggesting that it is lung-produced PROS1 rather than systemic PROS1 that might regulate disease severity [24].

PROS1 is an agonist for tyrosine kinase receptors MERTK and TYRO3, through which it limits the activation of innate immune responses, vascular permeability, response to cell damage, and coagulation-related pathologies in COVID-19 [25, 26]. The dysregulation of blood coagulation during COVID-19 results in depleted circulating PROS1 levels [27], which can consequently contribute to cytokine storm by reducing the immunosuppressive action of MERTK in macrophages [28, 29] and diminishing intrinsic PROS1 anticoagulant function [23, 25, 27, 30]. Subsequently, thrombo-inflammation can activate the complement system, enhancing inflammation in the lungs of COVID-19 patients [31]. SARS-CoV-2 can directly activate the complement system via all three complement pathways [31], and single-nucleotide variants of complement C4BP-α, which interacts with PROS1 [23], are risk factors for morbidity and death in COVID-19 [32].

However, the exact functions of PROS1 in regulating virus-induced responses in epithelial and myeloid cells locally in the human lung are largely unknown. In this study, using SARS-CoV-2-infected air–liquid interface (ALI) cultures of human primary bronchial epithelial cells and monocytes, we established that PROS1 reduces the epithelial and myeloid inflammation, promotes basal cell regeneration, and enhances antigen presentation, which may determine milder lung pathologies in viral diseases like COVID-19.

## Results

### SARS-CoV-2 infection of bronchial epithelium grown on air–liquid interface cultures limits the epithelial responses of COVID-19 lungs

**Figure 1.**
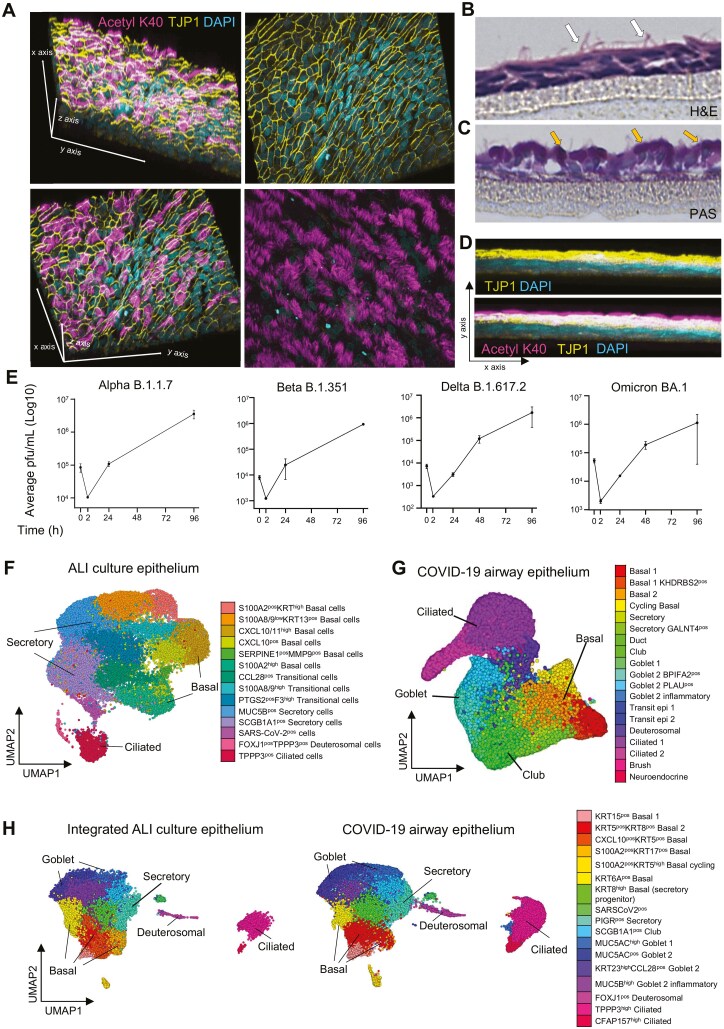
Air–liquid interface cultured epithelium as a system capable of replicating SARS-CoV-2 infection of human bronchial tissues. (A) Formation of pseudo-stratified epithelium with tight junctions (yellow) and cilia (magenta) in ALI cultures. Representative images of *N* = 4 independent staining. (B) H&E staining of pseudo-stratified epithelial transverse section with cilia (white arrows) in ALI cultures. 20X zoomed in. Representative images of *N* = 3 independent stainings. (C) PAS staining of the pseudostratified epithelium showing the mucin in red-purple (yellow arrows) in ALI cultures. 20X zoomed in. Representative images of *N* = 3 independent stainings. (D) Lateral view of the 3D section of epithelium from confocal microscopy Z-stacks, showing TJP1 layer (yellow) and the cilia (magenta) of ALI cultures. (E) Infection of the epithelial ALI cultures with different strains of SARS-CoV-2. The 0h represents the virus in the inoculum, the 2h represents the virus in the first wash of epithelium after incubation with the inoculum, and timepoints 24–96h measure the virus that was produced and released from the cells over time. Each dot represents the mean pfu/mL of 3 replicates with SEM. (F) UMAP visualization of 36 640 epithelial cells from ALI cultures. Each cell is represented by an individual point and is coloured by cluster identity. Clustering pipeline was run as described in the methods, and UMAP (RunUMAP) was generated for the chosen dimension of 25 and resolution of 0.5 (FindNeighbors, FindClusters). (G) UMAP visualization of 183 923 cells from lungs of healthy and COVID-19 patients published dataset, containing [33]. Each cell is represented by an individual point and is coloured by cluster identity as described in the published study [34]. (H). Split UMAP visualization of ALI cultured epithelia (*n* = 36 640 cells) data integrated with published airway epithelia dataset (*n* = 63 319 cells) with samples from nasal, tracheal, and bronchial epithelium of healthy donors (*N* = 7) and patients with mild (*N* = 5) to severe (*N* = 8) SARS-CoV-2 infection, subsetted from G [33].

To study the function of PROS1 in the regulation of virus-induced immune responses in human lung, we first established SARS-CoV-2 infection of ALI cultures. Figure 1A–D and Supplementary Fig. 1 demonstrate that ALI cultures result in the successful differentiation of pseudostratified bronchial epithelium with tight junctions, containing ciliated and goblet cells. We then established that different SARS-CoV-2 variants (Alpha B.1.1.7, Beta B.1.351, Delta B.1.617.2, Omicron BA.1) could readily infect and replicate in ALI epithelial cells, with viral load increasing progressively from 24 to 72 hours post-infection (Fig. 1E). Since our aim was to study whether PROS1 limits lung pathology, we used the more virulent Delta variant in the subsequent experiments [35, 36]. To evaluate if SARS-CoV-2-infected ALI culture resembles SARS-CoV-2 infection of human lungs, we performed single-cell RNA sequencing (scRNAseq) of control and Delta-variant infected cultures and integrated it with scRNAseq data set of lung tissue of healthy donors and patients with COVID-19 [33]. The scRNAseq of ALI cultures (Fig. 1F) identified different types of epithelial cells expected to be found in the upper airway and included distinct clusters of basal cells, transitional (an intermediate between basal and terminally differentiated), secretory (goblet), deuterosomal, and ciliated cells [10, 37, 38]. The ALI scRNA-seq dataset integrated well with human lung COVID-19 data, confirming that our SARS-CoV-2 ALI-infected system closely mimics the cells, their phenotypes, and responses observed in the upper airways of the human lung infected with the virus (Fig. 1G and H; Supplementary Fig. 2). In the subsequent experiments, we annotated ALI clusters with the identity derived from their integration with human lung tissue to facilitate translation [33] (Fig. 1F–H; Supplementary Fig. 2D).

### PROS1 is stored in basal cells and is secreted upon SARS-CoV-2 infection

**Figure 2.**
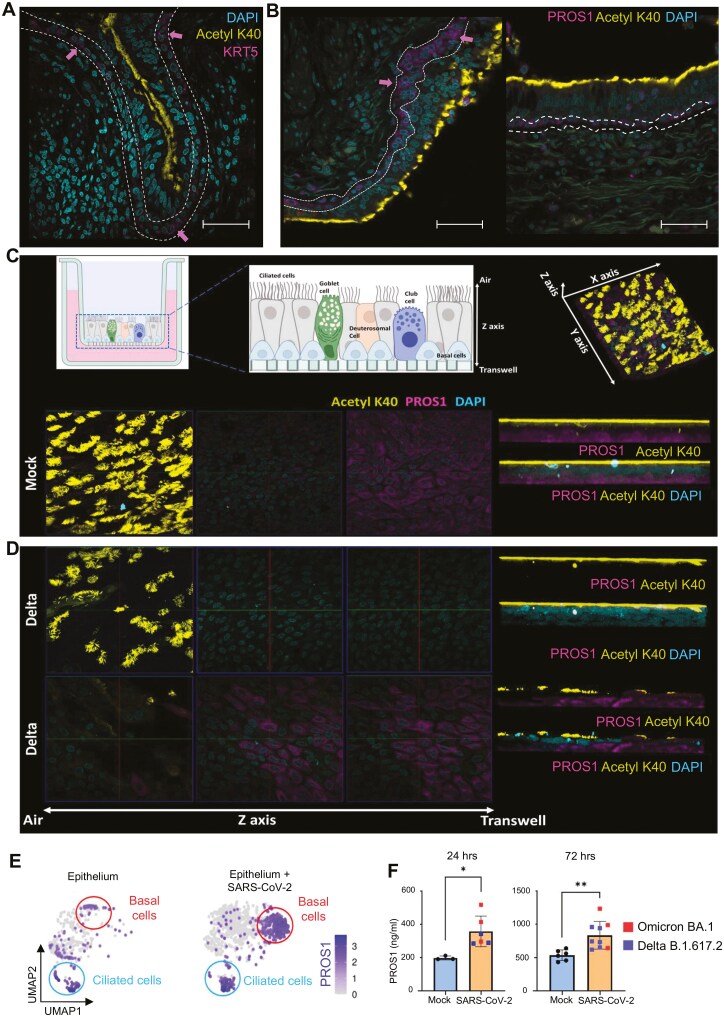
PROS1 is expressed in basal cells of pseudostratified epithelium and secreted upon SARS-CoV-2 infection. (A) Basal cells expressing KRT5 are found at the base of pseudostratified epithelium. 63X magnification, scale 50 μm. White line and pink arrows indicate basal layer expressing KRT5. (B) PROS1 expression at the base of pseudostratified epithelium. 63X magnification, scale 50 μm. White line indicates basal layer expressing PROS1. (C) PROS1 in ALI cultured bronchial tissue. Diagram of the Confocal Z scanning of the healthy ALI pseudostratified epithelium. In Mock (healthy) epithelium, PROS1 (magenta) was detected in the basal cell layer close to the transwell membrane. 63X magnification Z stack. (D) PROS1 detection (magenta) in SARS-CoV-2 (delta strain) infected epithelium across the *Z*-axis. Infection caused the loss of PROS1 from the basal cells. However, in infected epithelia, PROS1 could be detected in areas that had received cilia damage. Right: the lateral view of the 3D scanned tissues corresponding to the ortho-split images on the left. Z stack was performed at 63x magnification and analysed using the Zeiss Zen Black software. (E) Log-normalized expression of PROS1 visualized on UMAP embeddings of ALI epithelium and ALI epithelium infected with SARS-CoV-2. The highest expression of PROS1 was observed in basal cell clusters (red circle) and ciliated cells (blue circle). (F) Quantification of the secreted PROS1 in mock vs infected epithelium at 24 and 72 hours post-infection with SARS-CoV-2. PROS1 was secreted early upon infection from the epithelium and was elevated 72 hours after infection. Each group is represented by multiple replicates that were used as control or infected with SARS-CoV-2: Mock 24 hours *N* = 3, SARS-CoV-2 infected epithelia 24 hours, *N* = 6, Mock 72 hours, *N* = 6, SARS-CoV-2 infected epithelia 72 hours, *N* = 9. Data are presented as a bar plot with mean ± SD. Statistical comparison was performed using unpaired *t*-test between different conditions. *P < .05, **P < .01, ***P < .001, ****P < .0001.

To understand the role of PROS1 in the regulation of SARS-CoV-2 responses in the upper airways, we first investigated its expression in lung and ALI cultures. In healthy pseudostratified epithelium, PROS1 protein was mostly detected in the basal, KRT5-expressing cells (Fig. 2A and B). Similarly, in the *in vitro* ALI cultures, PROS1 was expressed in the basal cell layer close to the transwell membrane (Fig. 2C). At 72 hours following infection with SARS-CoV-2 Delta, PROS1 protein was largely undetected in the basal epithelial cells or moving upwards through the pseudostratified tissue (Fig. 2D)

To confirm that the loss of PROS1 protein in basal cells during infection was a result of its rapid release rather than inhibition of mRNA expression, we interrogated our transcriptomic data. We found that basal cells expanded upon virus infection, and those cells expressed high levels of PROS1 mRNA (Fig. 2E). At the mRNA level, we also observed the presence of PROS1 in the ciliated cells.

To test whether the cellular loss of PROS1 protein is due to its secretion by the basal cells, we evaluated PROS1 concentration in the media of infected and control ALI cultures at 24- and 72-hour post-infection (Fig. 2F). The data showed that infection resulted in increased PROS1 concentration in media from infected cells at both timepoints (Fig. 2F), explaining the absence of PROS1 detection in the basal cells by immunofluorescence (Fig. 2D).

In summary, SARS-CoV-2 infection triggers the release of stored PROS1 protein from the epithelial cells, elevating PROS1 level in the extracellular space.

### Basal cells express PROS1 receptors (MERTK/TYRO3) and shift toward a pro-regenerative phenotype in response to PROS1 during SARS-CoV-2 infection

**Figure 3.**
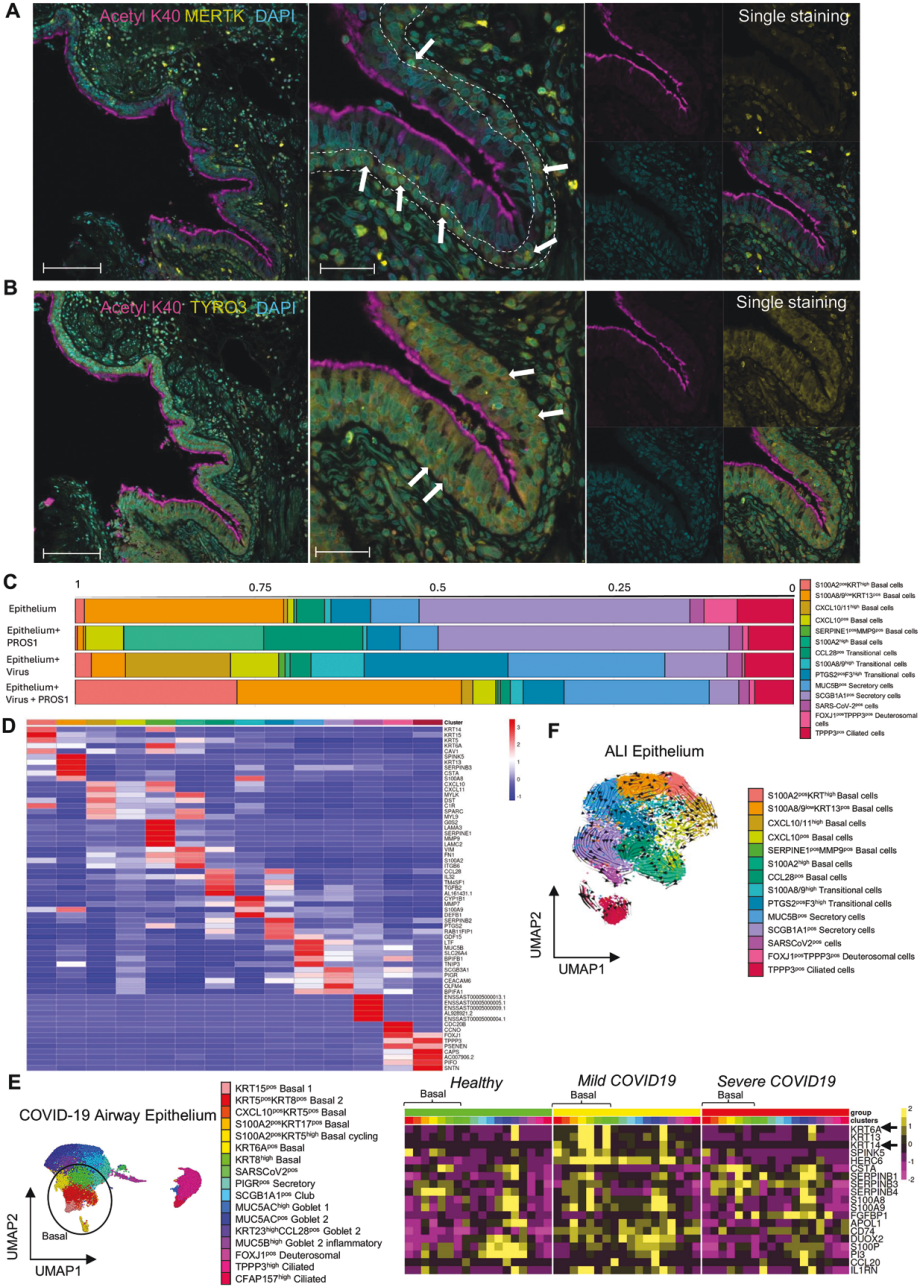
PROS1 acts on basal cells that express MERTK and TYRO1 and ameliorates their proinflammatory phenotype during SARS-CoV-2 infection. (A) Left: Expression of MERTK (yellow) in healthy pseudostratified epithelium. 20X magnification, scale = 100 μm. Middle, Z stack image projection, 63X magnification, scale = 50 μm. Right, split channels for Acetylated tubulin (cilia), DAPI (nuclei), and MERTK. Arrows indicate basal cells expressing MERTK. (B) Left: Expression of TYRO1 (yellow) in healthy pseudostratified epithelium 20X magnification, scale = 100 μm. Middle, Z stack image projection, 63X magnification, scale = 50 μm. Right, split channels for Acetylated tubulin (cilia), DAPI (nuclei), and MERTK. (C) Proportions of cell clusters in control ALI cultures at 72 hours, cultures stimulated with PROS1, cultures infected with SARS-CoV-2, and cultures infected with SARS-CoV-2 and treated with PROS1 dissected by scRNAseq. The proportion of cell clusters per each condition were obtained by generating a data frame (as.data.frame), using the epithelial object as for idents, and splitting by the SampleID metadata column that represented each condition (Supplementary Table 1). (D) Heatmap displaying the top five DE genes of each cluster in the ALI cultures, selected with a minimum log fold threshold of 0.25. The viral genes are displayed with their ENS Fasta ID, and their products are described in Supplementary Table 9. (E) Heatmap illustrating the scaled expression of the top 20 genes upregulated by PROS1 in virus-infected ALI cultures in COVID-19 airway epithelium. Gene expression values are illustrated as pseudobulk (average) expression values per cluster, per condition. KRT14 and KRT6A (markers of PROS1-stimulated epithelium) were increased in basal cell clusters (red circles) in patients with mild COVID-19. (F) Single-cell trajectory of cultured epithelial cells with RNA Velocity analysis visualized on UMAP embeddings. The direction of arrows infers the path of cell trajectory based on spliced versus unspliced RNA counts and suggests a differentiation path from CXCL10/11^high^ basal cells, which were increased during infection, to S100A2^pos^ KRT^high^ basal cells, characterizing infected cultures treated with PROS1.

We next investigated the expression of PROS1 receptors MERTK and TYRO3 in the pseudostratified epithelium (Fig. 3A and B). Immunofluorescent staining of healthy tissue showed expression of MERTK in the basal cells (Fig. 3A) and TYRO3 in all the pseudostratified epithelium (Fig. 3B), indicating that the secreted PROS1 could have autocrine effects on the airway cells.

To investigate the impact of PROS1 on SARS-CoV-2-infected pseudo-stratified epithelium from upper airways, we added PROS1 to ALI cultures that were infected with SARS-CoV-2 or mock-treated. The scRNAseq analysis of ALI cultures showed that SARS-CoV-2 infection increased the frequency of mucin-producing goblet cells, which is characteristic of SARS-CoV-2 infection of ALI cultures [38]. The viral-induced MUC5B^pos^ secretory cells, in addition to expressing *MUC5B* that is involved in COVID-19 pathology [34], also expressed genes indicative of viral and proinflammatory signatures (Fig. 3D). For example, these cells expressed mRNA encoding for *CSF3* and neutrophil chemoattractant *CXCL1, CXCL6, CXCL5*, and *CXCL8*, complement system protein *C3, CXCL17,* which is a chemoattractant for monocytes and dendritic cells, MHC class I genes (*HLA-A, HLA-C, HLA-E,* and *B2M*), and multiple IFN-induced genes (Fig. 3D and Supplementary Table 2). At protein level, CSF3 (G-CSF) was elevated in infected epithelial cultures at 72 hours (Supplementary Fig. 3D), whereas CXCL8 (IL-8) was elevated in infected cultures at 24 and 72 hours (Supplementary Fig. 3J), potentially originating from these secretory cells.

A novel observation of our study was the identification of SARS-CoV-2-induced proinflammatory phenotypes of basal cell (the CXCL10/11^high^ cluster) and transitional cells (PTGS2^pos^F3^high^ and S100A8/9^high^ clusters) (Fig. 3C and D and Supplementary Table 2).

**Figure 4.**
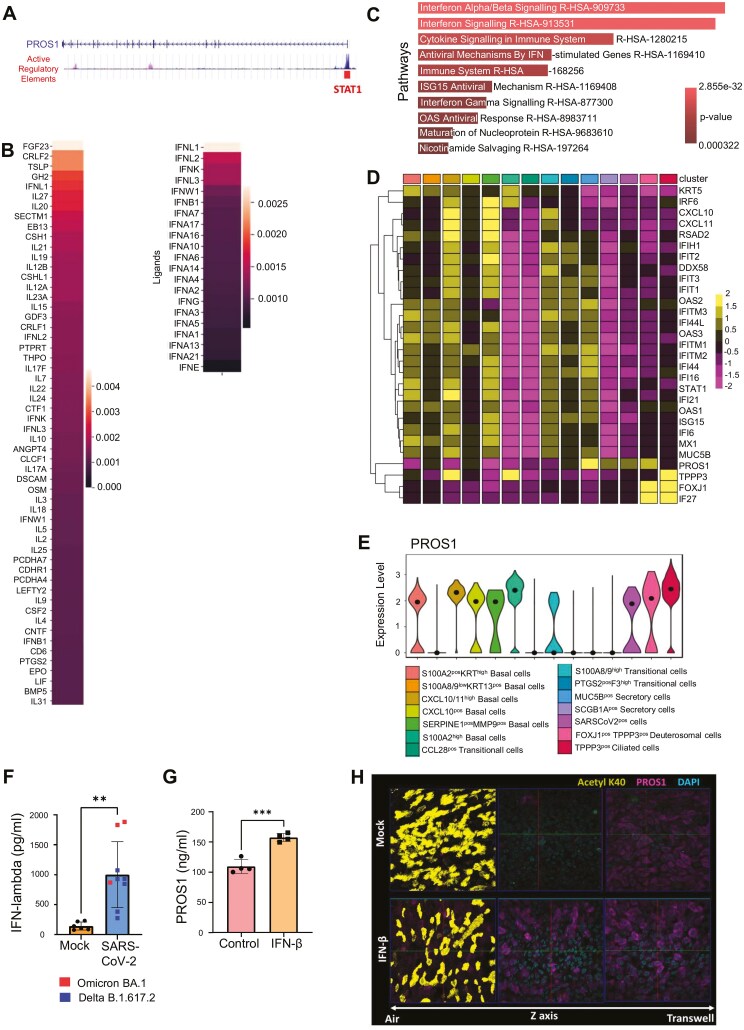
PROS1 is regulated by interferon signalling. (A) UCSC Genome Browser visualization of PROS1 gene, the H3K27Ac regions are indicated as active regulatory elements. Multiple transcription factors are reported to bind to PROS1 promoter (ORegAnno annotation), including the STAT1 (indicated), which is involved in interferon signalling and viral responses. Other transcription factors identified included: GATA2, TFAP2C, SMARCA4, CEBPA, TRIM28, CTCF, E2F4, ETS1, FOXA1, FOS, GATA3, HNF4A, TRIM28, MITF, RBL2, SPI1. (B) Multiple proteins are predicted to regulate the PROS1 production in epithelial cells. This was done using the ligand-receptor expression analysis (NicheNet), on an object containing only the epithelial cells from the sequenced BALF dataset [9]. Among multiple proteins that could potentially regulate PROS1 expression, either the top regulators (56 ligands with score >0.001) or type I and type III interferons are shown (score 0.001–0.003). (C) Visualization of top 10 reactome pathways, ordered by *P*-value ranking, of epithelial cells infected with virus. Reactome pathway activity inferred by evaluation of differentially expressed genes upregulated in epithelial cells infected with virus versus all other conditions with a minimum log.fc of 0.25 and a *P*-value <.05 based on Wilcoxon Rank Sum Test. (D) Average-expression heatmap visualizing scaled expression of selected DE IFN-inducible genes, alongside putative cluster marker genes, and PROS1 in ALI cultured epithelial cell clusters. DE marker genes for each cluster with a minimum log.fc of 0.25 were identified based on Wilcoxon rank-sum test × test with a *P*-value <.05 number (Supplementary Table 2). IFN-inducible genes were selected from this list (Supplementary Table 2). (E) Violin plots illustrating alra-imputed expression values of PROS1 in ALI cultured epithelial cell clusters. Black dot represents median expression value. (F) Infection of epithelium with SARS-CoV-2 resulted in Interferon lambda 1 secretion 72 hours after infection. Each group is represented by multiple replicates that were used as control or infected with SARS-CoV-2, Mock N = 6, SARS-CoV-2-infected epithelia, *N* = 9. Statistical comparison was performed using unpaired *t*-test between different conditions. *P < .05, **P < .01, ***P < .001, ****P < .0001. (G) Stimulation of ALI epithelial cultures with 10 ng/mL IFN-β for 24 hours upregulated PROS1 release. Each group is represented by multiple replicates that were used as control or stimulated with IFN-β, N = 4. Statistical comparison was performed using unpaired *t*-test between different conditions. *P < .05, **P < .01, ***P < .001, ****P < .0001. H. Epithelial ALI cultures were stimulated with IFN-β for 24 hours and then cultured without IFN-β for another 72 hours. The PROS1 (magenta) in IFN-β pre-stimulated cells at 72 hours was present in ciliated cell (Middle *Z* axis), as compared to the control cultures, where it was limited to the basal cells on the surface of the transwell (Left *Z* axis). PROS1 (magenta), Acetylated tubulin (yellow), DAPI (blue).

The cluster of CXCL10/11^high^ basal cells (KRT5^pos^) was characterized by high expression of *CXCL10* and *CXCL11* (Fig. 3D). These cytokines are mostly induced by type II IFN; however, since our ALI-infected epithelial cultures contained no added immune cells, their upregulation could be induced by type I IFNs [39]. In fact, multiple IFN-inducible genes were upregulated in sthis cluster (Supplementary Table 2 and Fig. 4D). In addition, this cluster expressed *DDX58* encoding for retinoic acid–inducible gene-I (RIG-I), and *IFIH1* encoding for melanoma differentiation-associated protein 5 (MDA5), which are pattern-recognition receptors for RNA viruses, and downstream induce type I/III IFNs (Supplementary Table 2) [40, 41]. It has been shown that RIG-I expression levels in human lung cells are important for the cellular defence in the initial SARS-CoV-2 infection, by restraining SARS-CoV-2 replication in a type I/III IFN-independent manner [42]. The CXCL10/11^high^ basal cells also had upregulated mRNA encoding for complement components (*C1R* and *C1S*), MHC class I (*HLA-A, HLA-B, HLA-C,* and *B2M*), and metalloproteases MMP2 and MMP13, the latter reducing repair during viral infection in bronchial epithelial cells (Supplementary Table 2) [43].

The PTGS2^pos^F3^high^ transitional cells were characterized by expression of the secretory marker *KRT23* [44], *PTGS2* encoding for cyclooxygenase-2, *PTGES* encoding for prostaglandin E synthase, coagulation factor III (*F3*), and plasminogen activator inhibitor 2 (*SERPINB2*), which all may play roles in COVID-19 inflammatory and coagulation symptoms. This cluster also expresses *EDN1* encoding for endothelin-1, a protein implicated in multiple lung diseases, and in endothelial and cardiovascular dysfunction in severe COVID-19 [45, 46]. Other interesting, upregulated genes of this cluster of cells include the components of tight junction claudin proteins (*CLDN1, CLDN2, CLDN7*), neutrophil chemoattractant (*CXCL1, CXCL5,* and *CXCL8*), MHC class I (*HLA-A, HLA-B, HLA-C, HLA-E,* and *B2M*), *IFIH1* encoding MDA5 for RNA virus detection, and multiple IFN-inducible genes (Supplementary Table 2).

S100A8/A9^high^ transitional cells were characterized by high expression of genes encoding for inflammation-inducing alarmins *S100A8* and *S100A9*. These epithelial cells can be responsible for the elevated S100A8/A9 observed in severe cases of SARS-CoV-2 infection [5, 47]. These transitional cells did not express *KRT5* as a differentially expressed gene but expressed *KRT6A* and *KRT6B* (Fig. 3D and Supplementary Table 2), which are stress-induced cytokeratins in airway basal cells with profibrotic phenotypes [47, 48]. These cells also expressed other upregulated genes characteristic of SARS-CoV-2 infection that were shared with CXCL10/11^high^ and PTGS2^pos^F3^high^ cells, such as *CXCL11, PTGES, IFIH1*, complement *C1R* and *C1S*, MHC class I encoding genes (*HLA-A, HLA-B, HLA-C, HLA-E, B2M*), metalloproteases *MMP14, MMP7,* and *MMP13*, tight junction protein Claudin 1 (*CLDN1*), multiple IFN-inducible genes, and multiple genes encoding ribosomal proteins (Supplementary Table 2).

Interestingly, PROS1 added to SARS-CoV-2-infected ALI cultures strongly downregulated the proportions of these SARS-CoV-2-induced phenotypes and favoured the emergence of a S100A2^pos^ KRT^high^ basal cell cluster (Fig. 3C). This cluster was characterized by the high expression of cytokeratin genes *KRT14*, *KRT15*, *KRT5,* and *KRT6A* (Fig. 3D), which are important for the differentiation of basal cells into secretory and ciliated cells (e.g. KRT14), and clonogenicity of basal cells (e.g. KRT15) [49]. This suggests the role of this cluster in the regeneration and repair of airway epithelium [49]. In the ALI/ COVID-19 human lung integrated data sets, his cluster integrated well with KRT^high^ cycling basal cells, further supporting their involvement in the regeneration of damaged epithelium (Supplementary Fig. 2C and D). We also demonstrated that the KRT14 and KRT6A expression was elevated in basal cells from patients with mild COVID-19 but not in healthy or severe COVID-19 patients (Fig. 3E), supporting the potential role of the KRT14^pos^ basal cells in mild disease phenotype.

RNA velocity analysis that infers cell trajectory from the direction of increased ratio of unspliced to spliced RNA counts showed that S100A2^pos^KRT^high^ regenerative phenotypes of basal cells differentiate from the SARS-CoV-2-induced inflammatory CXCL10/11^high^ basal cells (Fig. 3F). In addition, S100A2^pos^KRT^high^ showed expression of multiple genes that indicate the growth of these cells under SARS-CoV-2 infection conditions, such as those encoding IFN-induced proteins, S100 proteins (*S100A2, S100A11, S100A10, S100A8, S100A9, S100A14*), and MHC class I (*HLA-A, HLA-B, HLA-C, B2M*) (Supplementary Table 2). This suggests that PROS1 transforms the proinflammatory virus-induced basal cell phenotypes into pro-repair and regeneration phenotypes.

The addition of PROS1 alone to ALI cultures also affected two minor clusters. PROS1 reduced FOXJ1^pos^ TPPP3^pos^ deuterosomal cells and induced a small cluster of CXCL10^pos^ basal cells at comparable levels to viral infection. This similar effect observed in these two clusters between PROS1-stimulated controls and infected ALI cultures could be due to PROS1 secretion by the epithelium during infection (Fig. 2E).

In summary, these results suggest that local higher levels of PROS1 characterizing mild COVID-19 [24] might contribute to the reduced proinflammatory response and enhanced repair of the epithelial barrier in those patients.

### Interferon regulates PROS1 expression and secretion from the bronchial epithelium

After establishing that PROS1 is secreted from the epithelium upon infection with SARS-CoV-2, limiting inflammation and potentially driving regeneration of damaged epithelium, we sought to understand the mechanism that drives PROS1 expression and production during infection.

Knowing that viral infection induces IFN response from epithelium, we began by analyzing transcription factors downstream of IFN signalling that could bind the PROS1 promoter. Using the curated database of regulatory elements (ORegAnno) [50], we found that STAT1 was a significant regulator of PROS1 by binding its promoter region (Fig. 4A and Supplementary Table 5).

Furthermore, analysis of upstream regulators of PROS1 expression in airway epithelium [7], using NicheNet [51], predicted type I and type III IFNs among the top regulators of PROS1 (Fig. 4B and Supplementary Table 7).

Pathway analysis of scRNAseq data of ALI cultures using reactome [52] demonstrated that IFN pathways were significantly upregulated in SARS-CoV-2-infected ALI cultures, as compared to the mock-treated controls (Fig. 4C and Supplementary Table 6). Furthermore, our data showed that the basal cell clusters, which were the most affected by SARS-CoV-2, had significantly higher expression of IFN signalling genes (Fig. 4D), which was associated with higher *PROS1* mRNA levels (Fig. 4E). Thus, these results indicate that IFN signalling might be a driver of PROS1 mRNA expression in epithelial cells.

To validate the role of SARS-CoV-2 and IFN in the regulation of PROS1 expression, we investigated PROS1 protein expression and secretion in SARS-CoV-2-infected or IFN-stimulated ALI cultures. We found that type III IFN-λ1 was elevated in the medium of cells at 72 hours post-infection (Fig. 4F), confirming active production of IFNs by our ALI cultures upon viral infection that could affect PROS1 secretion (Fig. 4B).

To confirm that the IFN could induce PROS1 secretion, we stimulated epithelial cells for 24 hours with IFN-β. We used type I IFN-β because of its well-established role in regulating severe COVID-19, compared to type III IFNs [9, 18]. Despite their different effects on COVID-19 prognosis and their use of distinct receptor complexes, in cells, both type I and type III IFNs induce activation of STAT1, STAT2, and STAT3, as well as a similar subset of IFN-responsive genes [53]. The IFN-β stimulation resulted in higher levels of PROS1 in the media of stimulated cells compared to the controls, indicating IFN signalling induces early secretion of PROS1 (Fig. 4G).

To validate that IFN signalling activation, even at short term, would result in higher PROS1 expression in pseudostratified epithelium, we pre-stimulated ALI cultures with IFN-β for 24 hours, then cultured them for 72 hours without IFN (Fig. 4H). This was done to avoid long-term stimulation with IFN-β at concentrations used in cell culture, which is detrimental for cell survival [54]. We demonstrated that IFN-β pre-stimulation resulted in elevated PROS1 in both the basal cells and the ciliated cells that differentiate from basal cells, as compared to the mock-treated controls, where PROS1 was located only in the basal cell layer of the pseudostratified epithelium (Fig. 4H).

Together, these observations confirmed that IFN pathway activation results in early upregulation of PROS1 expression and secretion from the bronchial epithelium.

### Monocytes acquire the PROS1 receptor MERTK upon coculture with epithelium through an M-CSF-dependent mechanism

**Figure 5.**
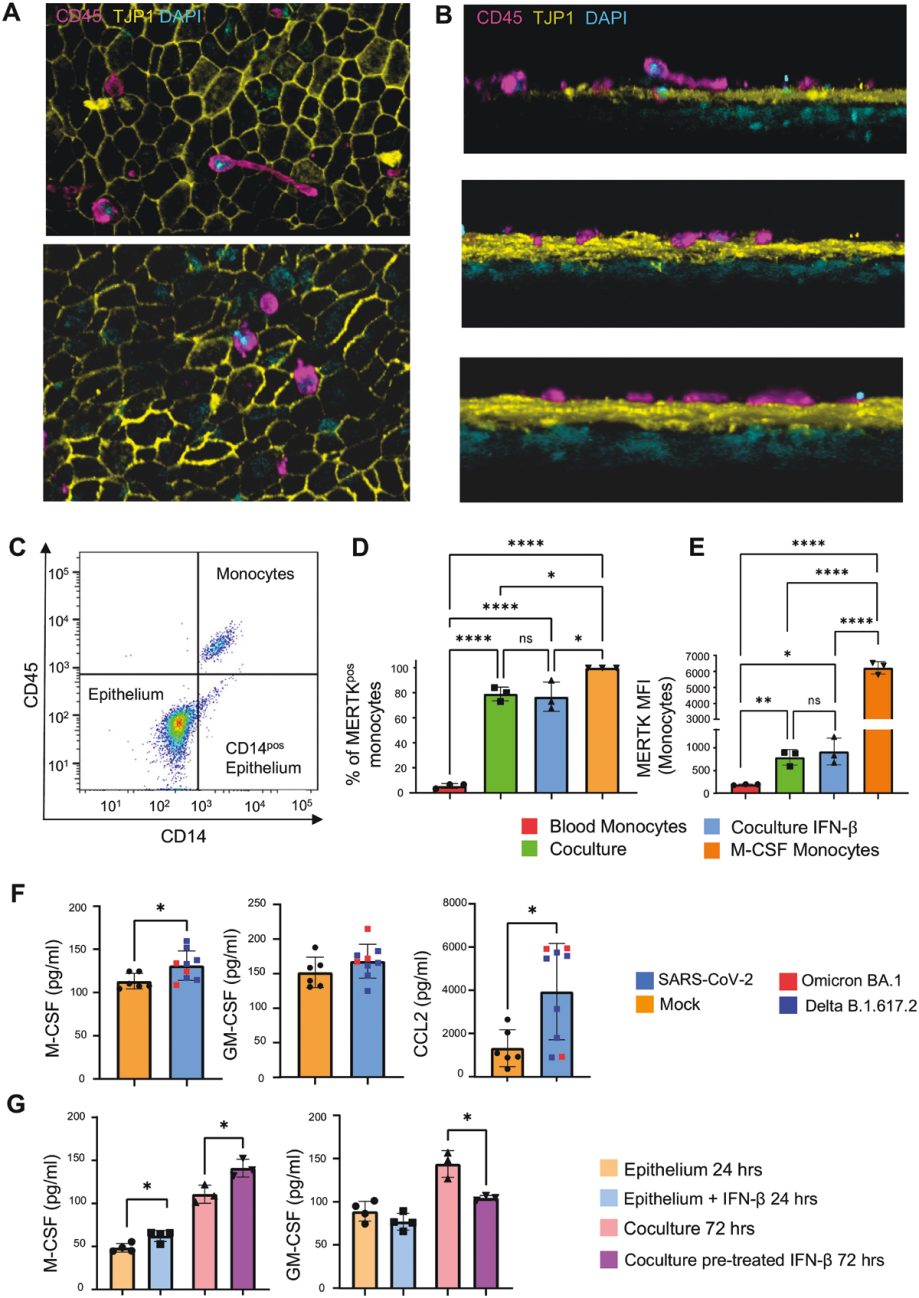
Monocytes acquire MERTK upon interaction with epithelium in ALI cultures. (A) Confocal 3D scans showing the monocytes on top of the epithelial barrier, at 72 hours co-cultures of ALI epithelia with monocytes. B. Lateral view of the 3D scan showing the monocytes on top of epithelial cells, not passing through the tight junctions. (C) Dot plot illustrating that monocytes can be isolated from co-cultures with ALI epithelium by expression of CD45 and CD14. (D, E) Upon contact with epithelium blood monocytes increased MERTK expression, as illustrated by % of MERTK-positive cells (D) and MFI of MERTK (E). Each dot represents monocytes from 3 different donors on 3 different ALI epithelial cultures (*N* = 3). For the positive control of MERTK expression, monocytes were cultured for 3 days with 50 ng/mL M-CSF. No M-CSF was used in co-cultures to allow assessment of MERTK expression only in response to epithelial-derived M-CSF. Statistical analysis was performed using one-way ANOVA with Tukey’s multiple comparisons test. *P < 0.05, **P < 0.01, ***P < 0.001, ****P < .0001. Data is presented as a bar plot ±SD of mean. (F) SARS-CoV-2-infected bronchial cells produced M-CSF and CCL2, but no GM-CSF, at 72 hours. Each group is represented by multiple replicates that were used as control or infected with SARS-CoV-2, Mock N = 6, SARS-CoV-2 infected epithelia *N* = 9. Data is presented as a bar plot with ±SD of mean. Statistical comparison was performed using unpaired *t*-test between different conditions. *P < .05, **P < .01, ***P < .001, ****P < .0001. (G) The IFN-β-stimulated epithelium did not affect GM-CSF production. IFN-β pre-treated cocultures had lower levels of GM-CSF compared to controls at 72 hours (left graph). IFN-β stimulation resulted in higher M-CSF production from the epithelium at 24 hours. At 72 hours, the cocultures pre-treated with IFN-β also had higher M-CSF levels than the controls (right). Each group is represented by multiple replicates that were used as control or stimulated with 10 ng/mL IFN-β (24 hours stimulated cultures, *N* = 4, 72 hours cocultures, *N* = 3). Data are presented as a bar plot with mean ± SD. Statistical comparison was performed using unpaired *t*-test between different conditions. *P < .05, **P < .01, ***P < .001, ****P < .0001.

To investigate the effect of epithelium-derived PROS1 on the monocytes that accumulate in bronchial lumen upon viral infection, we performed cocultures of monocytes with ALI pseudostratified epithelium (Fig. 5A–C). We hypothesized that modulation of myeloid responses by PROS1 can protect against inflammation and thus modulate disease severity.

Confocal microscopy showed that the monocytes formed direct contact with the epithelial cells, migrating and exploring the epithelial barrier in the ALI cultures (Fig. 5A and B). The monocytes survived well and could be readily recovered after 3 days in cocultures (Fig. 5C). Flow cytometry analysis showed that while circulating monocytes did not express MERTK, up to 80% of the monocytes cocultured with epithelial cells for 3 days acquired MERTK (Fig. 5D and E). IFN pre-stimulation of epithelium did not affect MERTK expression on monocytes, while M-CSF-treated monocytes expressed MERTK at the highest levels (Fig. 5D and E).

CSFs are known regulators of MERTK expression on monocytes. It has been shown that M-CSF upregulated MERTK, whereas GM-CSF downregulates it [29]. We observed constitutive production of CSFs by the ALI cultures and increased M-CSF but no GM-SCF upon SARS-CoV-2 infection (Fig. 5F). The infected ALI cultures also had higher concentrations of CCL2, which is the primary chemokine for monocyte recruitment to the tissues (Fig. 5F).

We also demonstrated that, similar to infection, M-CSF was higher in cocultures that were pretreated with IFN-β, whereas GM-CSF was largely unaffected by IFN signalling (Fig. 5G).

Collectively, these results demonstrate that SARS-CoV-2 infection causes bronchial epithelium to produce cytokines that attract and support myeloid infiltrates and induce expression of the PROS1 main receptor MERTK on these cells.

### PROS1 reduces the SARS-CoV-2-induced pro-coagulation and complement-producing monocytes and favors phenotypes with high expression of THBS1 and MHC class II

Having shown that SARS-CoV-2 infection causes release of PROS1 from the epithelium, and that the monocytes acquire the PROS1 receptor MERTK in cocultures with epithelium, we then explored the effects of PROS1 on the SARS-CoV-2-activated monocytes. Since PROS1-MERTK signalling has been shown to drive a pro-repair macrophage phenotype [29], we hypothesized that this axis may regulate myeloid responses during SARS-CoV-2 infection.

**Figure 6.**
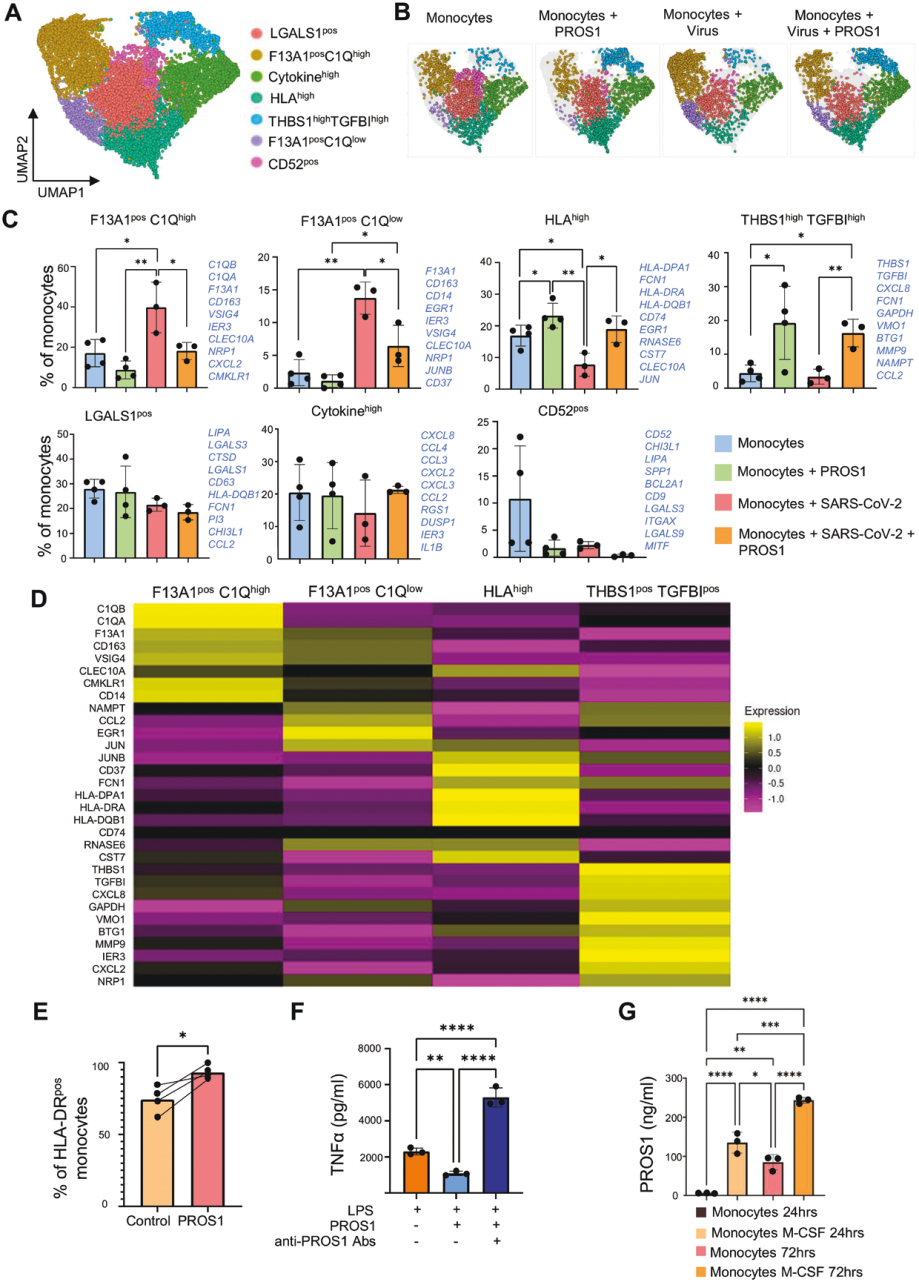
PROS1 downregulates the frequency of monocyte phenotypes with high expression of coagulation and complement genes, and upregulates phenotypes with MHC class II and THBS1 expression. (A) UMAP of monocytes cultured for 72 hours with 5 ng/mL M-CSF and in the presence of PROS1 (200 ng/mL), SARS-CoV-2 (10 000 pfu), or both. scRNAseq of cultures were performed with BD Rhapsody (*N* = 4 different donors). The whole object was normalized and integrated using Seurat SCTransform and the principal component analysis was run. The PCA embeddings were utilized for UMAP generation. The monocytes were subset (9839 cells), the PCA were used to determine the k-nearest neighbours for each cell during SNN graph construction before clustering at the chosen dimension of 22 and resolution of 0.3. (B) Split UMAP showing differences in proportion of cell clusters between conditions. (C) Changes in the proportion of distinct monocyte phenotypes between multiple conditions are presented as a bar plot with SD of the mean. Proportion of cells clusters as percentage of total cells. Due to the small number of cells for D1 (Monocytes + SARS-CoV-2 + PROS1) and D2 (Monocytes + SARS-CoV-2), these were removed from analysis, as proportions could not be obtained accurately. For this reason, a statistical comparison was performed using unpaired *t*-test between conditions. *P < .05, **P < .01, ***P < .001, ****P < .0001. (D) Pseudobulk heatmap showing the average expression of DE genes in the four clusters that were affected by PROS1 or SARS-CoV-2. Using the integrated monocyte object (Seurat SCT method), the assay is changed from SCT to RNA. The clusters of interests were subsetted from the rest into a new object. A list of top 10 significant genes (FindAllMarkers, minimum log.fc of 0.25, Wilcoxon Rank Sum Test, adj. *P* < .05) from each cluster was created, including the following genes in order: *C1QB, C1QA, F13A1, CD163, VSIG4, CLEC10A, CMKLR1, CD14, NAMPT, CCL2, EGR1, JUN, JUNB, CD37, FCN1, HLA-DPA1, HLA-DRA, HLA-DQB1, CD74, RNASE6, CST7, THBS1, TGFBI, CXCL8, GAPDH, VMO1, BTG1, MMP9, IER3, CXCL2, NRP1. A*verage expression was performed in the subsetted clusters object (AverageExpression). The heatmap was generated using the object with average expression of genes, and the list of the genes above (DoHeatmap). (E) Expression of HLA-DR by CD14 monocytes cultured for 72 hours in media with 5 ng/mL M-CSF and in media with 5 ng/mL M-CSF and PROS1 (200 ng/mL). Values are representative of four donors (*N* = 4). Statistical comparison was performed using paired *t*-test. *P < .05, **P < .01, ***P < .001, ****P < .0001. (F) TNF-α secretion upon overnight stimulation with LPS (1 ng/mL), LPS (1 ng/mL and PROS1 (200 ng/mL) or LPS 1ng/mL + PROS1 200ng/mL and anti-PROS1 PS7 antibody (10 μg/mL). Macrophages were differentiated for 6 days with 50 ng/mL M-CSF). For stimulation as above, the M-CSF was removed from the media. Data presented as a bar plot of three independent experiments (*N* = 3), with SD of the mean. Statistical analysis performed using one-way ANOVA with Tukey’s multiple comparison test. *P < .05, **P < .01, ***P < .001, ****P < .0001. (G) PROS1 (pg/mL) secretion from monocytes without and with M-CSF (50 ng/mL) at 24 and 72 hours of culture. Values are representative of three independent experiments (*N* = 3). Statistical analysis performed using one-way ANOVA with Tukey’s multiple comparison test. *P < 0.05, **P < .01, ***P < .001, ****P < .0001.

Single-cell RNA analysis of monocyte cultures incubated with SARS-CoV-2 revealed two monocyte phenotypes that were significantly increased in the presence of SARS-CoV-2 (i.e. F13A1^pos^ C1Q^high^ and F13A1^pos^ C1Q^low^ monocytes), as compared to mock-incubated cultures (Fig. 6C). These clusters were characterized by coagulation and complement gene signatures, in line with the phenotypes uncovered in patients with severe COVID-19 [6, 55, 56]. Both these clusters had expression of *F13A1*, encoding for fibrin-stabilizing coagulation factor 13 alpha chain. Coagulation is a feature of COVID-19, and it has been shown that consumption of the coagulation factor XIIIA (F13A1) was elevated in COVID-19 patients and tended to be higher in nonsurvivors [57]. These two clusters also expressed C1Q genes, albeit at different levels (Fig. 6D). Complement C1QA and C1QB have been shown to be expressed by the monocytes and macrophages in patients suffering from COVID-19 [6, 55], and these genes have been associated with complement activation and endothelial dysfunction that is observed in severe COVID-19 (56).

Adding PROS1 reduced the virus-induced pathogenic phenotypes (Fig. 6A–D) and increased monocyte clusters that were characteristic of PROS1 stimulation (i.e. HLA^high^ and THBS1^high^ TGFBI^high^ monocytes) (Fig. 6A–D).

The THBS1^high^TGFBI^high^ cell cluster had higher expression of mRNA encoding thrombospondin and TGF-β-induced protein. Thrombospondin plays a critical role in efficient wound repair in the lung via the activation of latent TGF-β (reviewed in [58]). The expression of the *TGFBI* on these cells could indicate autocrine TGF-β response, which goes parallel to the THBS1 expression. Thrombospondin also contributes to adequate repair by promoting IL-10 production and efferocytosis of apoptotic neutrophils by the macrophages [59]. Expression of these genes suggests a role of the PROS1-induced THBS1^high^TGFBI^high^ cells in the repair of the damaged and inflamed epithelial tissue, though this remains to be elucidated.

The other PROS1-induced HLA^high^ monocyte cluster was characterized by the expression of genes encoding for proteins involved in antigen presentation, such as MHC class II (*HLA-DPA1, HLA-DRA, HLA-DQB1, CD79*). In addition, this cluster expressed neutrophil recruiting chemokine *CXCL8* and SARS-CoV-2 entry receptor *NRP1*. The proportion of these cells was elevated for PROS1 supplementation alone and in the presence of SARS-CoV-2 and PROS1, but not in the presence of virus alone (Fig. 6C), showing that the MHC Class II gene upregulation is a strong feature of PROS1 signalling in monocytes regardless of viral infection.

Together, the above results indicate that during SARS-CoV-2 infection, PROS1 switched monocyte phenotypes away from pro-coagulation and complement-producing phenotypes toward antigen-presenting phenotype. To confirm this, we evaluated MHC class II expression in monocytes stimulated with PROS1 for 72 hours by flow cytometry. In accordance with the single-cell mRNA data, PROS1 stimulation of the monocytes resulted in cells with higher MHC class II as compared to unstimulated cells (Fig. 6E). Upregulation of MHC class II can enhance myeloid T-cell communication, allowing for efficient antiviral and effector functions [60, 61].

We further assessed the effects of PROS1 on the inflammatory response of monocytes. The monocyte-derived macrophages were stimulated overnight with LPS, in the presence or absence of PROS1 and PROS1-inactivating antibody PS7. PROS1 reduced TNF production from LPS-activated macrophages that was abrogated by the presence of PROS1-blocking PS7 antibody (Fig. 6F).

We then assessed the endogenous PROS1 production by the monocytes and upon their stimulation with M-CSF (Fig. 6G). Monocytes alone did not produce PROS1, but monocytes differentiating toward macrophages, either with M-CSF or media containing FCS, produced PROS1 (Fig. 6G). These results suggest that in lung tissue, infiltrating monocytes would first be exposed to exogenous, epithelial-derived PROS1, followed by exposure to endogenously produced PROS1 due to M-CSF released by epithelial cells.

In summary, these data suggest that PROS1 regulation of coagulation, complement activation, and pro-inflammatory activation of myeloid cells, as well as promoting their communication with effector T cells, may be the mechanisms by which PROS1 limits pathogenic myeloid cell responses during SARS-CoV-2 infection and protects against severe COVID-19.

## Discussion

Cellular mechanisms that promote mild pathology to SARS-CoV-2 infection over severe disease remain unexplored. We had previously observed higher PROS1 *mRNA* in the epithelium of patients with mild, compared to those with severe COVID-19 (24). Hypothesizing that PROS1 protects against severe COVID-19, in this study, we aimed to investigate the mechanisms by which PROS1 could modulate the epithelial and monocyte responses toward SARS-CoV-2 infection. We demonstrated that during SARS-CoV-2 infection, PROS1 modulates both epithelial and myeloid responses in the upper airway. In epithelial cells, the PROS1 strongly reduces proinflammatory phenotypes and changes them to pro-regenerative phenotypes that can repair the epithelial barrier. In the recruited monocytes, PROS1 reduces phenotypes with signatures of coagulation and complement, and upregulates MHC class II phenotypes that may communicate better with antiviral effector T cells. PROS1-induced reduction of epithelial-driven inflammation and promotion of epithelial barrier repair, combined with reduction of myeloid-driven coagulation and complement pathways and efficient antigen presentation, may all combine to determine a mild COVID-19 phenotype.

Using confocal microscopy and histology of healthy tissues, we showed that PROS1 is located in the basal cells of pseudostratified epithelium. PROS1 was absent in the SARS-CoV-2 Delta variant-infected cultures, with the exception of cells in areas lacking cilia, where PROS1 was expressed strongly. These areas could be most affected by the virus, as SARS-CoV-2 Open Reading Frame 10 (ORF10) protein induces the loss of cilia on epithelial cells by promoting their ubiquitin-dependent degradation [62]. Expression of PROS1 in these affected areas may provide protection against rapid disruption of the epithelium, as PROS1 has been shown to protect against epithelial apoptosis [63].

We then demonstrated that PROS1 concentrations in the media of infected cultures were higher than in the controls, indicating elevated secretions of PROS1 upon infection. This suggested that PROS1 was stored in basal cells and constitutively secreted in healthy airways, but during SARS-CoV-2 infection, PROS1 was rapidly secreted from the basal cells.

In order to assess potential autocrine effects of epithelium-secreted PROS1, we stained for PROS1 receptors MERTK and TYRO3 (29) and found that the healthy epithelial cells express both receptors. The expression of these receptors could also explain the protective roles that PROS1 exerts on the epithelium [63]. The expression of MERTK was visibly higher in basal cells than in the ciliated cells. To investigate the role of PROS1 in epithelial biology upon SARS-CoV-2 infections, we used SARS-CoV-2-infected ALI system that we validated to mimic SARS-CoV-2 infection of the human lung [33].

SARS-CoV-2 infection affected the secretory cells and the basal cell populations of the ALI system. This included an increase in the proportion of mucin and cytokine-producing secretory cells (MUC5B^pos^ cluster), while no difference was observed in the ciliated cells, in accordance with published results from SARS-CoV-2-infected ALI cultures [38]. However, we did observe that in the SARS-CoV-2-infected epithelium, unlike in the controls, regions of the pseudostratified epithelium were lacking or had a little cilia, as described above. Post-translational effects on cilia formation due to infection [62] would not necessarily be caught at the transcriptome level, or it could also be that the infected ciliated cells were lost upon digestion of tissue. The MUC5B^pos^ cells were not affected by the addition of PROS1 in culture, showing that their phenotype is strongly characteristic of SARS-CoV-2 infection.

This study also identified three novel phenotypes of basal and transitional cells, which were upregulated for SARS-CoV-2 infection: the CXCL10/11^high^ basal cells, PTGS2^pos^F3^high^ transitional cells, and the S100A8/A9^high^ transitional cells. Mediators produced by these cells are positively associated with COVID-19 severity. In COVID-19, markedly elevated CXCL10 levels are related to ARDS and neurological complications, such as the loss of smell and taste, and are a good biomarker of disease severity [64–66]. Prostaglandin E2 (PGE2) levels are positively correlated with COVID-19 severity [67], whereas F3 levels are positively correlated with cell senescence and hypercoagulation in COVID-19 patients [68]. Metalloproteases are associated with COVID-19 pathology, such as the MMP13 that reduces repair during viral infection in bronchial epithelial cells [43, 69].

Interestingly, we observed that these proinflammatory epithelial cells were strongly reduced by PROS1. This demonstrated the importance of PROS1 in modulating epithelial-derived phenotypes and mediators of SARS-CoV-2 infection, thus modulating the severity of COVID-19. Furthermore, PROS1 caused the conversion of the CXCL10/11^high^ basal cells to a S100A2^pos^KRT^high^ basal cell phenotype, which was characterized by expression of cytokeratin genes involved in regeneration and repair of the epithelial barrier [49]. By integrating our SARS-CoV-2-infected ALI cultures with a dataset of COVID-19 patients [33], we also demonstrated that the KRT14 expression was elevated in basal cells from patients with mild but not in severe COVID-19, indicating that the KRT14^pos^ basal cells are important for mild disease phenotype.

Having shown that PROS1 is secreted upon SARS-CoV-2 infection from the bronchial epithelium and its effects on epithelial cell phenotypes during infection, we further investigated mechanisms that regulate PROS1 secretion. IFN signalling is a primary antiviral response against SARS-CoV-2 [9, 17–19], and was the primary signature in our infected ALI cultures. Multiple IFN-induced genes were significantly elevated in the epithelial cells captured by the single-cell transcriptomics, especially in the basal cells described above.

Type I IFNs are crucial for the successful defence against SARS-CoV-2 and mild COVID-19, while impairment of their production results in severe disease [17–19]. Furthermore, efficient initiation of type III IFN production in the upper airways can lead to rapid elimination of the SARS-CoV-2 and limit viral spread to the lower airways [9]. Localization of IFN response is also important in the lung with regard to disease severity. It has been shown that SARS-CoV-2 induces the efficient production of IFN-III (IFN-λ1 and IFN-λ3) in the upper airways of younger and/or milder patients, whereas critically ill patients express high levels of IFN-I and IFN-λ2 in the lower airways [9]. When assessing the IFN production from our infected epithelium, we observed a cumulative secretion of type III IFN, but no type I (Supplementary Fig. 2G–I). This could be because our epithelial culture systems originated from cells of a younger individual, who tend to produce a type III response against SARS-CoV-2 in the upper airway [9].

We found that STAT1 binds to the promoter region of PROS1 (50) and further demonstrated that IFN-β stimulation of epithelial cells resulted in rapid secretion of PROS1 at 24 hours. Furthermore, pretreatment of epithelial cultures with IFN-β overnight to initiate IFN signalling resulted in elevated PROS1 in the ciliated cells at 72 hours. These results together showed that viral-induced IFN response is important for the PROS1 production and secretion.

We found that in addition to ameliorating SARS-CoV-2-induced pathogenic responses of the epithelium, PROS1 can also potentially limit SARS-CoV-2-induced inflammatory activation of monocytes recruited into the bronchial lumen. Monocytes infiltrate at an early stage of disease, and their interaction with epithelium has been shown to determine COVID-19 severity [20]. We observed that the infection of epithelial cultures resulted in elevated M-CSF that is known to upregulate PROS1 receptor MERTK on monocytes and macrophages [29]. We demonstrated that CD14+ monocytes acquired MERTK in cocultures with bronchial epithelial cells, due to the M-CSF produced by the epithelium. This meant that the monocytes, which lack MERTK in circulation, when infiltrating the upper airway, would be able to respond to PROS1 by expressing MERTK.

SARS-CoV-2 induced two pathogenic monocyte clusters, F13A1^pos^C1Q^high^ and F13A1^pos^C1Q^low^, with high expression of genes encoding proteins involved in coagulation and complement pathways that are associated with severe COVID-19 [6, 55–57]. These include the coagulation factor XIIIA (*F13A1*) that is elevated in COVID-19 patients and is higher in nonsurvivors [57], complement *C1QA* and *C1QB* that are associated with complement activation and endothelial dysfunction in COVID-19 (56), as well as proinflammatory cytokines and chemokines such as *CXCL8*, *CCL2, CXCL2*, and *MMP9* which impairs epithelial tight junction integrity and barrier functions [70].

Interestingly, PROS1 significantly inhibited these pathogenic clusters and induced potentially protective clusters of the THBS1^high^TGFBI^high^ and the HLA^high^ monocytes, expressing high levels of genes belonging to thrombospondin/TGF-β tissue-repair response [58, 59] and antigen-presenting pathways, respectively. The former, together with PROS1-induced activation of repair program in basal cells, might be key in inducing efficient lung tissue repair after clearance of pathogens. The increased expression of MHC class II was also confirmed at protein level upon PROS1 stimulation of monocytes. Upregulation of MHC class II can enhance myeloid-T cells communication, allowing for efficient antiviral and effector functions [60, 61]. T-cell responses, such as those including type 1 CD4^+^ T-cell phenotype (Th1), are crucial for effective viral control that results in mild COVID-19 (61).

### Study limitations and future directions

The study presents evidence that PROS1 release occurs following infection and coincides with a shift from proinflammatory to regenerative epithelial phenotypes. However, whether PROS1 is a cause or consequence (or both) of this shift remains to be elucidated in future studies. In addition, while this study showed that IFN signalling, predicted through STAT1, results in upregulation of PROS1 in the epithelial cell, the exact mechanisms remain to be elucidated in future work. Similarly, the mechanisms by which CSFs drove PROS1 upregulation in monocytes and the role of PROS1 in phagocytosis of apoptotic epithelial cells during infection remain to be elucidated in future work. These validation experiments would help to better differentiate between correlation and causation, and lead to unravelling of interesting cellular mechanisms characteristic of viral infections.

This study was not able to directly visualize the presence of virus in the epithelial populations undergoing phenotypic changes, nor in the PROS1-producing cells. Future studies could address this by employing immunohistological co-detection of viral proteins, PROS1, and the epithelial markers characterized here.

## Conclusion

Overall, in this study, we describe a novel natural mechanism by which the upper airway regulates inflammation against SARS-CoV-2 in COVID-19, and potentially in other viral diseases. PROS1 regulates inflammation by reducing proinflammatory activation of the epithelium and promoting pro-repair phenotypes. In parallel, PROS1 reduced myeloid phenotypes associated with severe COVID-19, while supporting a phenotype with efficient antigen presentation. Together, these effects counteract the cellular responses correlated to severe COVID-19 and thus may contribute to milder disease in patients. Modulation of PROS1 in viral disease can therefore be a therapeutic target for protection against severe inflammation and airway damage.

## Methods

### Donors and biological variance

The monocytes used in this study were collected from healthy individuals, listed in the Supplementary Table 4. To account for sex variance, experiments were designed so that the cells from donors used in experiments came from individuals of different sexes. Furthermore, the donors were selected to be over 45 years of age, as the severe inflammation during COVID-19 was more prominent in older individuals (Supplementary Table 4).

### Epithelial air–liquid interface culture

Human primary bronchial/tracheal epithelial cells (ATCC, PCS-300-010; Donor: Hispanic/latino male,14 years old), passage 2, were expanded in T25 flasks in Airway Epithelial Cell Basal Medium (ATCC, PCS-300-030), supplemented with Bronchial Epithelial Cell Growth Kit (ATCC PCS-300-040) and 10 U/mL Penicillin and 10 μg/mL streptomycin (Gibco, 15140-122). After the initial expansion, the cells were collected from the flasks and cultured on polycarbonate transwells (NUNC,140620, 0.4 μm pores, surface area 0.47 cm^2^), at a density of 1 × 10^5^ cells/cm^2^. Before adding the cells, the transwells were coated with 4.5 μg/cm^2^ Rat tail collagen I (Gibco, A10483-01) in distilled water at 37°C for 6 hours. Following this incubation, the transwells were washed twice with 1X D-PBS (Gibco, 14190-094) and dried at 37°C for another 15 minutes. The cells on transwells were incubated for 4 days, changing the expansion medium after 2 days.

After the expansion of cells in the transwells, the expansion media were removed, and the apical (cells) and basal compartments of the transwells were washed with warm D-PBS. For the differentiation of epithelial cells in ALI conditions, the Pneumacult ALI differentiation media was used, comprised Pneumacult ALI Basal Medium (StemCell Technologies, 05002) with 1X ALI Supplement (StemCell Technologies, 05003), and supplemented with 1% ALI maintenance Supplement (StemCell tech. 05006), 4 μg/mL Heparin (StemCell Tech. 07980), 0.48 μg/mL Hydrocortisone (StemCell Tech. 07925), and 10 U/mL Penicillin and 10 μg/mL streptomycin (Gibco,15140-122).

The ALI differentiation medium was added to the basal compartment, and cells were cultured at ALI for 4 weeks. The media was changed every 2 days. When the mucus production was visible, the cells were washed as needed with warm 1X D-PBS. Hydrocortisone was removed from the ALI differentiation medium 3 days before the infection and co-cultures. The experiments took place on Week 5 of ALI culture.

### Isolation of PBMCs and CD14 monocytes

The PBMCs were isolated from the blood of healthy donors using density separation using Histopaque-1077 (Sigma-Aldrich, 10771-500ML). The blood was diluted 1:2 with sterile 1X D-PBS (Gibco, 14190-094), and then slowly added on top of Histopaque-1077. The blood was centrifuged at 2500 RPM for 25 minutes at room temperature, without a break. After the centrifugation, the PBMCs were collected from the buffy coat. The PBMCs were used directly or stored overnight at 4°C before isolation of CD14 monocytes.

CD14 monocytes were isolated using the AutoMACS ProSeparator (Miltenyi Biotech, 130092545) and CD14 labelled beads (Miltenyi Biotech, 130050201), as described in the manufacturer’s protocol. The CD14-positive monocytes were kept on ice in RPMI-1640 media (Gibco, 21875-034), supplemented with 10% FBS (Sigma-Aldrich, F9665), 2 mM L-Glutamine (Sigma-Aldrich, G7513-100ML), and 100 U/mL Penicillin and 100 μg/mL streptomycin (Gibco, 15140-122), until ready to be cultured.

### Infection

SARS-CoV-2 England-02 (hCoV-19/England/02/2020, GISAID accession EPI_ISL_407073) was obtained from Public Health England. The Alpha (B.1.1.7), Beta (B.1.351), and Delta (B.1.617.2), variant isolates were kindly provided by Wendy Barclay (GISAID accession numbers EPI_ISL_723001, EPI_ISL_770441, and EPI_ISL_1731019, respectively). The Omicron B.1.1.529.1 (BA.1) variant was described previously (GISAID accession number EPI_ISL_10666879) [71].

Epithelial cell cultures were developed as described above, and cortisol was removed 3 days before infection. The cells were infected with 10 000 pfu of SARS-CoV-2 variants Delta B.1.617.2 and Omicron BA.1 in 100 μL DMEM (DMEM High Glucose GLUTAMax Pyruvate—Gibco 31966021). After 2-hour incubation at 37°C, the inoculum was collected, and the epithelial layers were washed once with 100 μL DMEM (DMEM High Glucose GLUTAMax Pyruvate—Gibco 31966021). The wash was collected as 2 hpi time point. At 24, 48, and 96 hpi, 100 μL of DMEM was added to the apical layers and incubated at 37°C for 30 minutes. After incubation, the cell washes were stored at −80°C. After all time points were collected, samples were assayed for virus level by plaque assay on Vero E6 F5 cells, which is a subclone of Vero E6 cells with enhanced susceptibility to SARS-CoV-2 [72].

The same assay was performed earlier to determine the infectivity of the epithelial cultures toward SARS-CoV-2 Alpha strain B.1.1.7 and Beta strain B.1.351.

### Co-culture of epithelium with CD14 monocytes

CD14 monocytes were added on top of epithelium after overnight infection with SARS-CoV-2. The cells were added as a drop of 6 μL containing 1 × 10^5^ cells on the surface of the epithelial cells. The density of the monocytes relative to the surface area of the epithelium on the transwell was 2.1 × 10^5^ cells/cm^2^. Some of the co-cultures were incubated in Pneumacult ALI maintenance medium without hydrocortisone, and some were incubated in the same medium supplemented with PROS1 at 200 ng/mL (Biotechne, 9489-PS). Monocyte-only controls were prepared by seeding 2.1 × 10^5^ cells/cm^2^ and incubated in complete RPMI medium (5 ng/mL M-CSF) or complete RPMI medium supplemented with PROS1 at 200 ng/mL. The co-cultures and controls were incubated at 37°C for 72 hours before single-cell RNA Extraction.

### Culture of monocytes with SARS-CoV-2

To study the direct effect of virus on immune cells, PBMCs from four donors were cultured with SARS-CoV-2 strain BetaCoV/England/02/2020/EPI_ISL_407073 for 72 hours. For this experiment, the cells were seeded at a 2 × 10^5^ cells/cm^2^ in 24-well plates and cultured for 72 hours in 400 μL of complete RPMI medium (with 5 ng/mL M-CSF) alone (Control), complete RPMI with 200 ng/mL PROS1, complete RPMI 10 000 pfu of SARS-CoV-2, and complete RPMI with 10 000 pfu of SARS-CoV-2 and 200 ng/mL PROS1. After the incubation period, the cells were collected for RNA extraction from single cells.

### BD Rhapsody single-cell extraction

On the days of the extraction, the cells were washed with D-PBS, which was collected in individual tubes. Then the cells were lifted using Triple Express (Gibco, 12604-013) for 15 minutes at 37°C. The cell suspension was then transferred into the respective tubes, and the transwells were washed once with D-PBS to collect all the cells. The dead cells were then removed using the EasySep Dead Cell Removal (Annexin V) Kit (StemCell Technologies, 17899) as described in manufacturer’s protocol, with the only difference that the cells were collected in 2 mL polypropylene tubes (Sarstedt, 72693) and live cells were negatively separated using DynaMag2 magnet (Life Technologies, 12321D), in order to avoid pouring of suspensions while working with the virus.

The cells were then tagged in FACS buffer using the BD Human multiplex tags (BD, 633781). After the incubation, the cells were washed three times, pooled together, and loaded onto the scRNA-seq BD Rhapsody Cartridge of single-cell RNA extraction, using the BD Rhapsody Cartridge Reagent Kit (no. 633731) according to the manufacturer’s protocol. In the final stage, cDNA was synthesized on mRNA captured on the beads using BD Rhapsody cDNA Kit (Cat. No. 633773), following the manufacturer’s protocol.

### Library formation, mapping, and analysis

Libraries of 399 genes were prepared using BD Rhapsody Targeted mRNA and the Tag Amplification Kit (BD 633774) and primers from the BD Rhapsody Immune response Panel Hs (399 genes, BD 633750), as per the manufacturer’s protocol.

Whole transcriptome libraries were prepared using the Whole Transcriptome Analysis and Sample Tag library Preparation kit (BD 633801), as per the manufacturer’s protocol (2019 version).

Sequencing was performed using Illumina system sequencing services by Glasgow Polyomics.

Mapping was performed on BD Seven Bridges Genomics website. For the target genes, Fastq files containing the raw sequencing were mapped against a reference panel (BD-Rhapsody_immune_Response_Panel_Hs.fasta). For whole transcriptome sequencing, Fastq files containing the raw sequencing were used to map against a reference genome (GRCh38-PhiX-gencodev29-20181205.tar.gz) with supplemental virus genome (Sars_cov_2.ASM985889v3.cds.all.fa), and transcriptome annotation (gencodev29-20181205.gtf). Sample Tag information is displayed in Supplementary Table 1.

### Analysis of epithelium single-cell transcriptomics

Analysis of the data has been performed using R version 4.1.0. The Seurat package v4 was used to create an object (CreateSeuratObject function) from the RSEC_MolsPerCell files generated from SevenBridges mapping. The data were normalized (NormalizeData), and the top variable genes were identified for all samples (FindVariableFeatures). The data were scaled (ScaleData), and principal component analysis was run (RunPCA).

#### Quality control and filtering.

Cell filtering involved removal of cells with less than 200 expressed genes and more than 2000 expressed genes, as well as cells expressing more than 40% mitochondrial genes (subset, subset = nFeature_RNA > 200 & nFeature_RNA < 2000 & nCount_RNA > 200 & nCount_RNA < 4000 & percent.mt < 40). This cleaning allowed for the exclusion of doublets and dying cells.

#### Clustering pipeline.

 The PCA embeddings were visualized and then used for UMAP generation (RunUMAP). The same PCs were used to determine the *k*-nearest neighbors for each cell during SNN graph construction before clustering at the chosen dimension of 26 and resolution of 0.2 (FindNeighbors, FindClusters).

#### *Cluster* id*entification and epithelial cluster subsetting.*

Clusters were identified by their expression of canonical epithelial marker genes (FeaturePlot) and identification of cluster-markers (FindAllMarkers). Epithelial cell clusters were separated into a new object from the co-cultures myeloid cells that were mostly duplets with epithelial cells, and a small cluster of fibroblasts with high expression of collagen genes (subset).

#### Epithelial analysis.

The data of the new epithelial object was normalized (NormalizeData) and the top variable genes were identified for all samples (FindVariableFeatures). The data were scaled (Scale Data), and the principal component analysis was run (RunPCA). The PCA embeddings were utilized for UMAP generation (RunUMAP). Clustering pipeline was run as described above, for the chosen dimension of 25 and resolution of 0.5 (FindNeighbors, FindClusters). The clusters and the analysis in the images of this paper are for these clusterings. Metadata columns are summarized in the Supplementary Table 1.

Clusters were visualized in UMAP graph (Dimplot) and upregulated genes per each cluster were identified (FindAllMarkers, log fold threshold 0.25). (Supplementary Table 2 with adjusted *P*-value of <.05).

#### Enrichr Reactome pathway analysis.

 Using the epithelial object from above, the idents (Idents) were set to SampleID metadata column, representing different culture conditions such as Epithelial, Epithelial + PROS1, Epithelial + SARS-CoV-2, and Epithelial + SARS + PROS1. The top upregulated genes for each group were obtained (FindAllMarkers, log fold change threshold 0.25) (Supplementary Table 6). The top genes were then used to analyze pathways using the Reactome 2022 in Enrichr website as previously described [52, 73–76], choosing to display only the statistically significant pathways.

Upstream regulators of PROS1 expression in airway epithelium were predicted NicheNet package [51]. Using the following script (https://github.com/saeyslab/nichenetr/blob/master/vignettes/ligand_target_signaling_path.md), ligand-to-target (PROS1) signaling paths were inferred, and potential ligands were organized by prediction score (Supplementary Table 7).

#### Monocyte analysis:

SCT Normalization (SCTransform) was used to normalize, scale, and find variable features. The sample datasets were integrated using SCT pipeline (SelectIntegrationFeatures, FindIntegrationAnchors), and the principal component analysis was run (RunPCA). The PCA embeddings were utilized for UMAP generation (RunUMAP). Monocyte clusters were separated into a new object (subset). Clustering pipeline was run as described above, for the chosen dimension of 22 and resolution of 0.3 (FindNeighbors, FindClusters). The clusters and the analysis in the images of this paper are for these clusterings. Markers were found on SCT assay (PrepSCTFindMarkers, FindAllMarkers with parameters min.pct = 0.4, logfc.threshold = 0.6, recorrect_umi = FALSE).

#### Cell proportion plots:

The proportion of cell clusters per each condition was obtained by generating a data frame (as.data.frame), using the epithelial object as for idents, and splitting by the SampleID metadata column that represented each condition (Supplementary Table 1). The data were visualized using the png and ggplot dev.off functions.

#### Pseudobulk heatmap.

 The average gene expression was computed per cluster using the AverageExpression function in Seurat. Columns were reordered based on predefined cluster order, and data were standardized. A cluster annotation dataframe was created, ensuring correct ordering and mapping of annotation colours. Finally, a heatmap was generated using pheatmap, maintaining cluster order and visualizing scaled expression values.

Using the integrated monocyte object (Seurat SCT method), the assay is changed from SCT to RNA (DefaultAssay). The clusters of interests were subsetted from the rest into a new object (subset). A list of the top 10 significant genes for each cluster is created, listing the genes in order as they would appear on the heatmap. Next, average expression was performed in the subsetted clusters object (AverageExpression). The heatmap was generated using the object with the average expression of genes, and the list of genes above (DoHeatmap).

### Cell trajectory analysis

Single-cell trajectory analysis (RNA velocity [77]) of cultured epithelial cells was performed by estimation of spliced and unspliced counts using the velocyto command line interface (velocyto run10x). Outputted.loom files for each culture, which contained transcript splicing data, were incorporated into the epithelial cell Seurat object by splitting the object by culture, loading each.loom file using the (ReadVelocity, SeuratWrappers (0.3.0)) and creating a new assay for spliced, unspliced, and ambiguous counts before merging our samples back together again, recreating our integrated Seurat object. This Seurat object was then converted for application in python (SaveH5Seurat, Convef using SeuratDisk (0.0.0.9019) package. The converted.h5ad file was then read into Python using the scanpy [78] (1.9.3) package, which creates an AnnData (0.9.1) object (sc.read). The spliced and unspliced count data were normalized and preprocessed as recommended by scvelo [77] (0.3.1) before running RNA velocity analysis. RNA velocities were estimated for each cell using the (scv.tl.velocity, scvelo), specifying deterministic modelling. Velocities were projected onto the pre-computed UMAP embedding using (scv.pl.velocity_embedding_stream, scvelo).

### Integration with COVID-19 airway epithelium

The generated cultured epithelial dataset was integrated scRNAseq data from nasal, tracheal, and bronchial airway epithelium of healthy donors and patients with mild to severe SARS-CoV-2 infection [33]. The data were obtained in.h5ad format from https://covid19cellatlas.org and were converted using SeuratObject (4.1.3) and SeuratDisk (0.0.0.9015). All pediatric samples were removed along with any cells not of epithelial cell lineage, resulting in a dataset of 63 319 cells. Ensemble geneIDs were converted to gene names using gprofiler2 (0.2.1, gconvert). These data were then integrated with cultured epithelial data with Harmony () integration. Datasets were merged based on the expression of 18 525 common genes, and the median number of counts per cell between both datasets was identified. This value was used to set the scaling factor for combined log-normalization of the merged data (NormalizeData, scale.factor = 1926). Feature selection was performed (FindVariableFeatures) and the data was scaled (ScaleData) prior to principal component analysis (RunPCA). Cell embeddings from the selected top 40 principal components (PCs) were used in UMAP generation (RunUMAP) to allow for visual inspection of batch separation prior to integration. Harmony [79] integration was then performed using the Seurat wrapper function (RunHarmony, SeuratWrappers, 0.3.0), specifying theta values for sample ID (theta = 1) and dataset (theta = 3). The resulting harmony-corrected PCA embeddings were then used for UMAP generation. The same PCs were used for SNN graph construction before clustering at the chosen resolution of 1 (FindNeighbors, FindClusters). Identified clusters were annotated by generating confusion matrix [80], visualized as a heatmap illustrating the proportion of cells from published airway epithelium reference clusters within each of the new clusters identified post-integration with cultured cells. Differentially expressed genes between clusters were identified (FindAllMarkers, test.use = MAST), and such cluster marker genes were identified as those with a significant adjusted *P*-value of <.05 (Bonferroni and multiple test correction) and expressed by greater than 40% of cells in the cluster (“min.pct” parameter 0.4).

### Transwell paraffin embedding

Epithelial cells were fixed in 8% formaldehyde in PBS overnight at room temperature (RT) and stored in D-PBS at 4°C. Before dehydration, the D-PBS was carefully removed from the transwell. The fixed cells were dehydrated by incubation with 35%, 50%, 70%, and 95% ethanol for 10 minutes each, and incubation in 100% ethanol twice, 10 minutes each. After the dehydration, the cells were infiltrated twice using Histoclear for 10 minutes each. Infiltration of cells was continued by removing the Histoclear and adding liquid paraffin to the bottom of the well and to the transwell insert. The transwells were incubated at 58°C for 1 hour. After this incubation, the paraffin was changed, and transwells were incubated for another hour. After the second incubation, the plates were put at 4°C surface for 20 minutes, to solidify the paraffin. The transwell was carefully removed from the well, and the membrane was cut using a small, sharp scalpel blade. The block containing the transwell membrane was then inserted into a paraffin boat as if it were a piece of tissue, an orientation that would allow transverse sections to be cut. After solidification, the paraffin blocks were kept at RT until cut for the histology.

### Hematoxylin and eosin staining of transwell paraffin sections

For histological staining, the transwells embedded in paraffin were cut into 5-μm sections. The sections were mounted on 1 mm SuperFrost Plus adhesion slides (Epredia, J1800AMNZ). The slides were allowed to dry and then inserted in an oven for 35 minutes at 60°C. This was followed by dewaxing in Xylene, twice for 3 minutes each. After that, the slides were hydrated slowly by incubation in 100%, 90% 70% ethanol, twice and for 3 minutes each. The hydration was completed by incubation in water for 5 minutes.

For hematoxylin and eosin (H&E) staining, the cells were incubated in Harris hematoxylin for 2 minutes and washed with water for 30 seconds. The cells were dipped twice in 1% acid/alcohol solution and rinsed with water. This was followed by incubation in Scott’s Tap Water Substitute for 30 seconds, and a quick rinse in running water. To counterstain, the cells were dipped in 70% ethanol 10 times, and then incubated in 1% eosin for 3 minutes. Following this incubation, the cells were dehydrated using 90% twice for 30 seconds and 100% ethanol twice for 3 minutes. The cells were incubated in Xylene, twice for 3 minutes each, and then cover slips were mounted using DPX.

### PAS (Periodic Acid Schiff) staining of transwell paraffin sections

The transwell sections were deparaffinized and hydrated to water as described above. The transwells were then oxidized in 0.5% periodic acid solution for 5 minutes. Following a rinse in distilled water, the slides were placed in Schiff reagent for 15 minutes. The slides were then washed in lukewarm tap water for 5 minutes, and counterstained in Mayer’s haematoxylin for 1 minute. The slides were washed in tap water for 5 minutes and dehydrated as described above. The cells were incubated twice in Xylene for 3 minutes each, and then cover slips were mounted using DPX.

### Immunofluorescent staining of ALI cultures on transwells

Virus-infected epithelial cells on transwells were fixed in 8% formaldehyde in PBS overnight at RT and stored in D-PBS at 4°C until ready to be stained. The cells were fixated with ice-cold 100% methanol and incubated overnight at −20°C. The following day, the methanol was removed, and transwells were stored dry at 4°C until ready for staining.

The cells were washed 3 times for 3–5 minutes each with D-PBS, and blocked for 1 hour at RT with rocking. Blocking buffer comprised of 1% BSA in 1X D-PBS, 2% Human Serum (Invitrogen, 31876), 2% Goat Serum (Invitrogen, 31872). For permeabilization during blocking, the blocking buffer was supplemented with 0.2% to 1% Triton ×100 for methanol- and PFA-fixated transwells, respectively. The cells were washed 3 times with D-PBS for 3–5 minutes, and the primary antibodies diluted in 1% BSA in D-PBS were added. The cells were incubated overnight at 4°C with rocking.

After the incubation with the primary antibody, the antibody solution was removed and the transwells were washed 4 times with D-PBS, 5 minutes each. Then the secondary antibodies were added on the transwells diluted in 1X D-PBS at a final concentration of 2 μg/mL. The transwells were incubated at RT for 2 hours, in the dark. Antibodies and their concentrations are summarized in Supplementary Table 8.

After the incubation, the transwells were washed 3X with D-PBS, and then cut into squares using a surgical scalper. The mounting of the transwell section was performed by adding 8 μL of VECTASHIELD DAPI mounting medium (2B Scientific, H-1800-10) on the slide, followed by the transwell membrane with the cells on top. Then another 8 μL of DAPI mounting medium was added on the top, and a coverslip was used to cover the cells. The slides were left to curate initially at RT for 2 hours, and then at 4°C overnight, before being observed using confocal microscopy at 63X oil immersion.

For the staining of monocytes in ALI coculture, the same technique as described above was used. Antibodies and their concentrations are summarized in Supplementary Table 8. Images were obtained using conformal microscope Zeiss LSM 880, and images were acquired using Zeiss Zen Black software.

### Immunofluorescent staining of healthy upper airway tissue.

Formalin-fixed paraffin-embedded 5-μm-thick upper airway sections were obtained from a healthy biopsy (7123/16). The slides were inserted in an oven for 35 minutes at 60°C. This was followed by dewaxing in Xylene, twice for 5 minutes each. The slides were hydrated by incubation in 100%, 90%, 70% ethanol, twice, and for 3 minutes each. The hydration was completed by incubation in distilled water for 5 minutes. Epitope retrieval was performed in 0.01 M citrate buffer pH 6, and heated using a microwave running on 50% power for 5 minutes, and at 30% power for 8 minutes, until boiling started. The samples were then washed in TBS with 0.025% Triton-X for 5 minutes each and blocked for 2 hours at RT in TBS with 10% human serum, 10% goat serum, and 1% BSA. After blocking, the slides were washed twice in TBS, and primary antibodies or isotopes were added. The slides were incubated overnight at 4°C. The following days, the slides were incubated for 2 hours at RT with the secondary antibodies. Then another 8 μL of DAPI mounting medium was added on the top, and a coverslip was used to cover the cells. The slides were left to curate initially at RT for 2 hours, and then at 4°C overnight, before being observed using confocal microscopy at 63X oil immersion. Antibodies and their concentrations are summarized in Supplementary Table 8.

### IFN-β Stimulation of ALI Epithelium

For IFN stimulation of the bronchial epithelial cells, hydrocortisone-free ALI differentiation medium was supplemented with 10 ng/mL IFN-β (Peprotech, 300-02BC). The old media was removed, and the IFNβ-supplemented media was added to the basal compartment of the transwells. The cells were incubated overnight, and the following day, the IFNβ-media was removed and collected for ELISA, and transwells were washed only in the basal compartment. Then, hydrocortisone-free ALI media was added to the transwells, and co-cultures were set immediately after as described above. As positive controls for MERTK expression, monocytes were seeded in 24-well plates (Corning, 3524), at a density of 1 × 10^5^ cells/cm^2^, in complete media supplemented with 50 ng/mL human M-CSF (Peprotech, 30025).

### Flow cytometry

On the days of the extraction, the cells were washed with D-PBS, which was collected in individual FACS tubes (Falcon, 352052). Then the cells were lifted using Triple Express (Gibco, 12604-013) for 15 minutes at 37°C. The cell suspension was then transferred into the respective tubes, and the transwells were washed once with D-PBS to collect all the cells. The macrophage controls were also collected using the same method. The cells were then labelled with the viability dye eFluor780 (Invitrogen, 65086514) in D-PBS for 20 minutes in the dark on ice. After the incubation period, cells were washed by adding 1 mL of FACS buffer and centrifugation at 1600 RPM for 5 minutes at 4°C. FACS buffer was comprised of 1X D-PBS with 2% FBS (Sigma-Aldrich, F9665), 2 mM L-Glutamine (Sigma-Aldrich, G7513-100ML), and 100 U/mL penicillin and 100 μg/mL streptomycin (Gibco, 15140-122). The supernatant was removed, and cells were labelled with a mix of antibodies against markers of interest for 30 minutes in the dark on ice. Antibodies and their concentrations are summarized in Supplementary Table 8. Unlabelled cells, cells labelled only with eFluor780 (Invitrogen, 65086514), and FMO for each marker of interest were used as technical controls for gating. The data acquisition was performed using BD LSR FORTESSA cytometer. The results were analyzed using FlowJo_v10.8.0.

### PROS1 assays on the monocyte-derived macrophages

CD14 monocytes were isolated immediately after PBMC isolation from the whole blood of healthy donors. The PBMCs were isolated as described above. The monocytes were cultured in complete RPMI medium supplemented with 50ng/mL M-CSF (Peprotech, 30025). The monocytes were differentiated by incubation in M-CSF supplement compete RPMI media for 6 days, changing the medium on Day 3. On Day 6, the cells were stimulated with 1 ng/mL LPS (Sigma L6529, clone 055:B5), or with 1 ng/mL LPS or 200 ng/mL PROS1 overnight, in the absence of M-CSF. Unstimulated and PROS1-stimulated cells were kept as controls. The supernatant for ELISA was collected after the overnight stimulation.

To test the effect of blocking endogenous and exogenous PROS1, we used the anti-PROS1 PS7 antibody (Santa Cruz Biotech, sc-52720) at 10 μg/mL, on macrophages stimulated by LPS (1 ng/mL) or LPS (1 ng/mL) and PROS1 (200 ng/mL) overnight.

### ELISA

The media from the lower compartment of the transwells of co-cultures and epithelia infected with SARS-CoV-2 were inactivated using a handheld UV Lamp (UVS-28 EI Series UV Lamp, 8 WATT, 254nm, P/N 95-0249-02, 0.32 AMPS, Analytik Jena GmbH). The lamp was placed on top of the plate, and the samples were exposed to UV twice for 2 minutes with a 2-minute break in between exposures. Inactivated medium was collected into centrifuge tubes and stored at −80°C until ready for ELISA. An aliquot of the supernatant was tested in infection assays for the presence of the virus before the supernatant was ready for ELISA.

The media from epithelial cells, co-cultures, and monocytes treated with IFN-β and the respective controls were collected into centrifuge tubes. The supernatants from cultures, where the media was in direct contact with the cells, were centrifuged at 3000 rpm for 10 minutes to ensure no cells were present. After centrifugation, the supernatant was carefully collected into new sterile 1.5-mL tubes, avoiding disturbance of the pellet. For transwells, the media from the lower compartment was not centrifuged, because the 0.4-μm pores would not allow cells to pass through the membrane.

Protein S was quantified using the human PROS1/Protein S ELISA Kit (AssayGenie, HUFI01701) as per the manufacturer’s protocol. TNF-α was quantified using a human TNFα ELISA kit (Thermofisher, CHC1753), as per the manufacturer’s protocol. CXCL8 was quantified using human IL-8 ELISA kit (Thermofisher, CHC1303), as per the manufacturer’s protocol.

The absorbance was measured using a plate reader (BioTek, ELx800), and the concentration was calculated using a standard curve generated from the reconstituted standards.

For quantification of multiple cytokines, GeniePlex Multiplex Immunoassay kits were used as instructed by the manufacturers (AssayGenie). The custom human 14-plex consisted of TNF-α, IL-1β, IFN-α2, IFN-β, IFN-λ1(CD29), IL-8 (CXCL8), M-CSF (CSF1), GM-CSF (CSF2), G-CSF (CSF3), Osteopontin (SPP1), Calprotectin (S100A8/A9), IL-6, CCL2 (MCP-1), and IL-10.

### Statistics

Detailed statistical methods are provided in each figure legend and in the scRNAseq method sections above. For single-cell RNA analysis, the default Wilcoxon rank-sum test was used to calculate the DEGs (Seurat, FindAllMarkers).

## Supplementary Material

kyaf012_suppl_Supplementary_Figures_1

kyaf012_suppl_Supplementary_Figures_2

kyaf012_suppl_Supplementary_Figures_3

kyaf012_suppl_Supplementary_Tables_1-9

## Data Availability

All scRNAseq data raw files are available from ArrayExpress under accession number (E-MTAB-14405). Data is available to readers upon request to the corresponding author.
