# Supplementary figures and images for: PROS1 released by lung basal cells limits inflammation in epithelial and monocytes during SARS-CoV-2 infection
Source: Discov Immunol. 2025 Aug 27;4(1):kyaf012. doi: 10.1093/discim/kyaf012 (PMC12448451; doi:10.1093/discim/kyaf012)

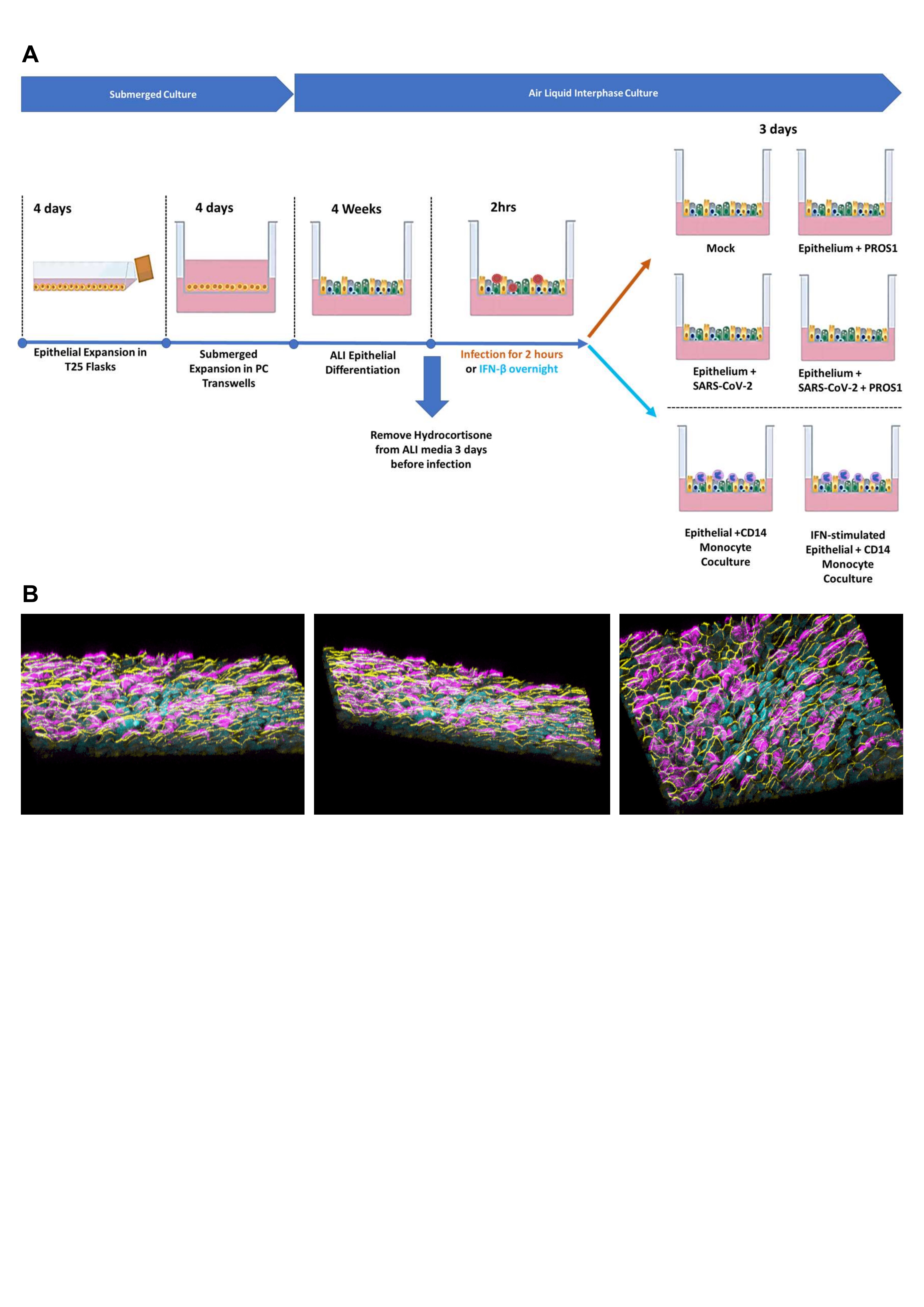

Supplement: kyaf012_suppl_Supplementary_Figures_1 [file kyaf012_suppl_supplementary_figures_1.jpeg]

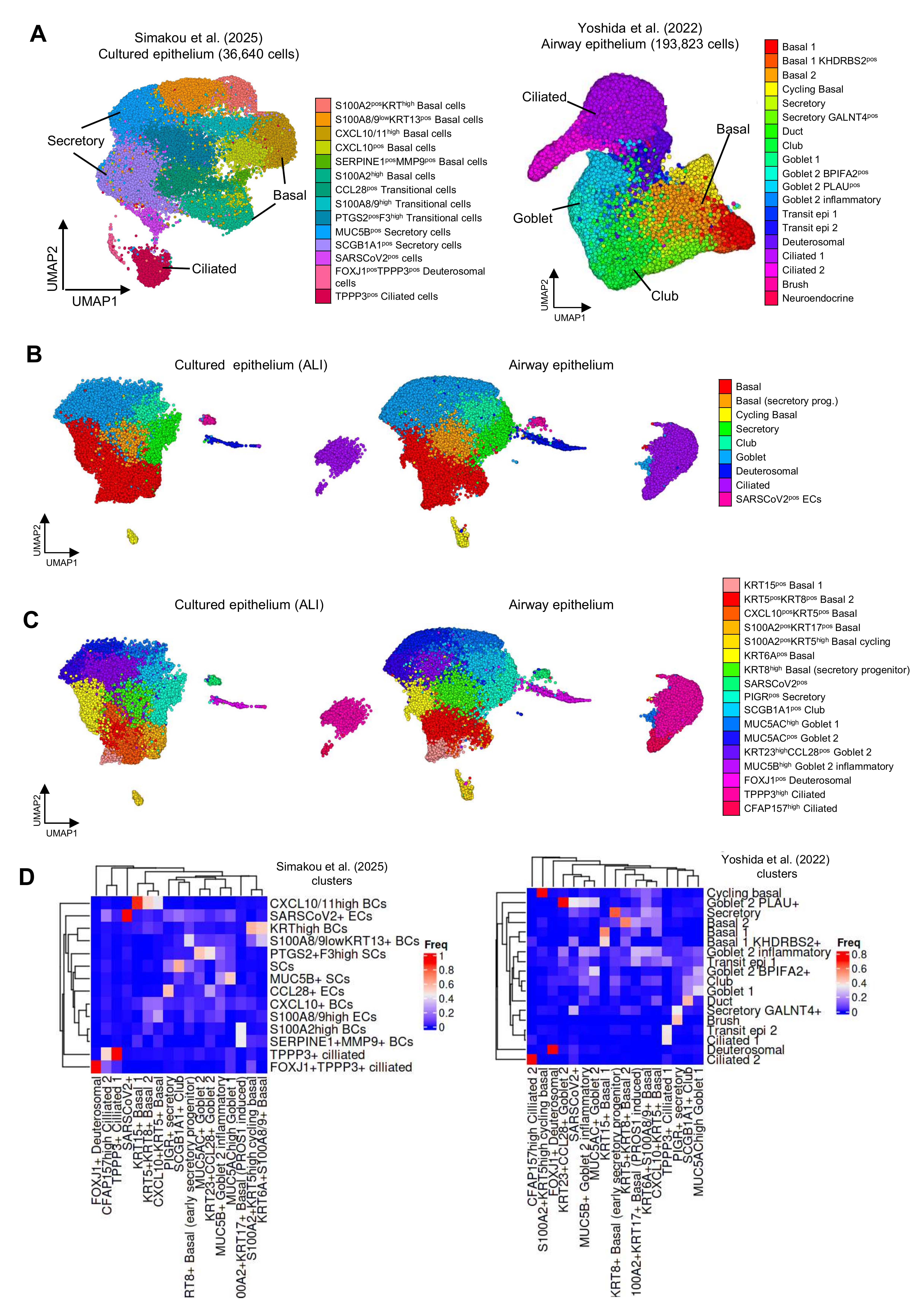

Supplement: kyaf012_suppl_Supplementary_Figures_2 [file kyaf012_suppl_supplementary_figures_2.jpeg]

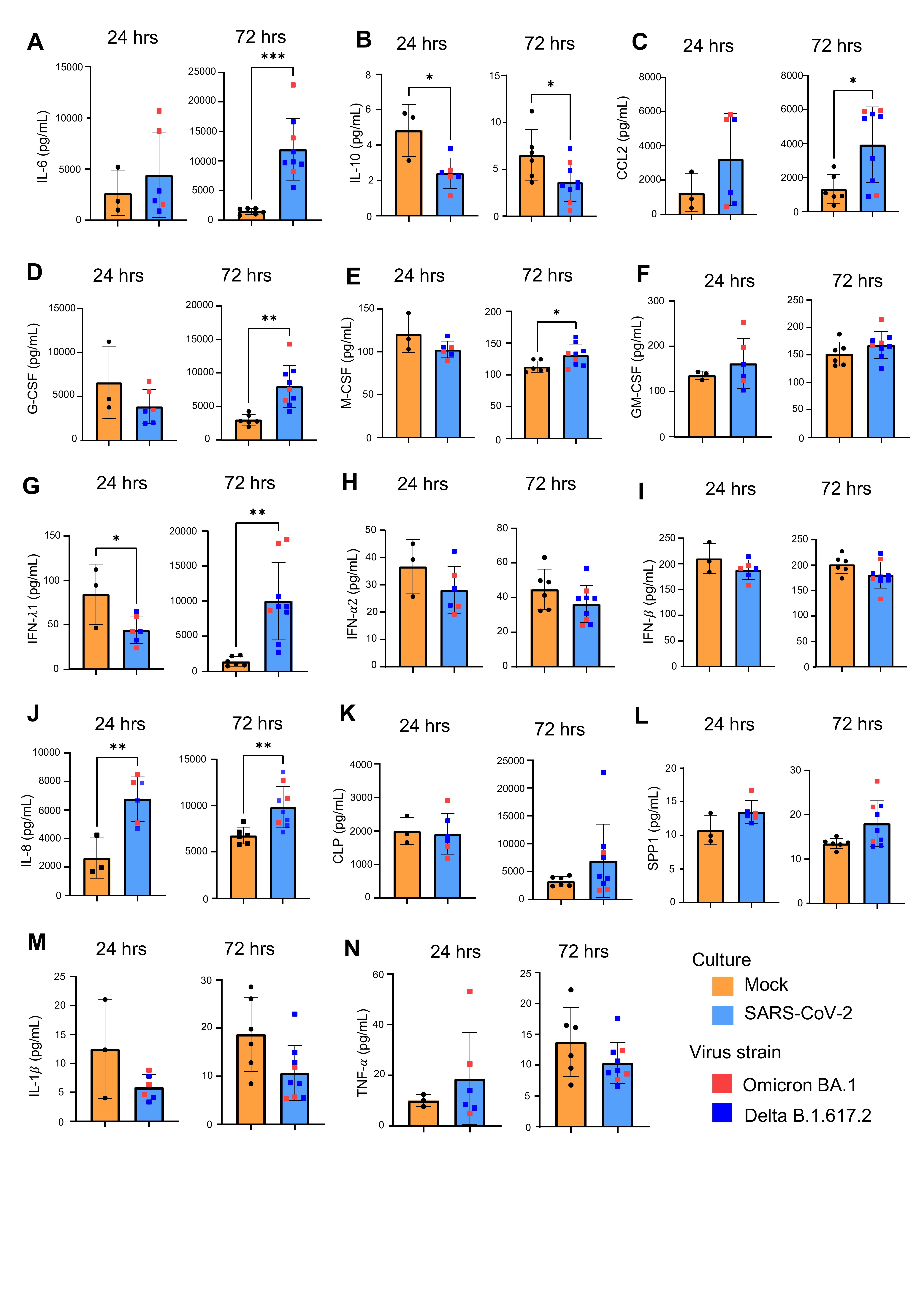

Supplement: kyaf012_suppl_Supplementary_Figures_3 [file kyaf012_suppl_supplementary_figures_3.jpeg]
